# The establishment of COPD organoids to study host-pathogen interaction reveals enhanced viral fitness of SARS-CoV-2 in bronchi

**DOI:** 10.1038/s41467-022-35253-x

**Published:** 2022-12-10

**Authors:** Louisa L. Y. Chan, Danielle E. Anderson, Hong Sheng Cheng, Fransiskus Xaverius Ivan, Si Chen, Adrian E. Z. Kang, Randy Foo, Akshamal M. Gamage, Pei Yee Tiew, Mariko Siyue Koh, Ken Cheah Hooi Lee, Kristy Nichol, Prabuddha S. Pathinayake, Yik Lung Chan, Tsin Wen Yeo, Brian G. Oliver, Peter A. B. Wark, Linbo Liu, Nguan Soon Tan, Lin-Fa Wang, Sanjay H. Chotirmall

**Affiliations:** 1 Lee Kong Chian School of Medicine, Nanyang Technological University, Singapore, Singapore; 2 Programme in Emerging Infectious Diseases, Duke-NUS Medical School, Singapore, Singapore; 3 School of Electrical and Electronic Engineering, Nanyang Technological University, Singapore, Singapore; 4 Department of Respiratory and Critical Care Medicine, Singapore General Hospital, Singapore, Singapore; 5 Duke-NUS Medical School, Singapore, Singapore; 6 Priority Research Centre for Healthy Lungs, Hunter Medical Research Institute and School of Medicine and Public Health, University of Newcastle, Newcastle, NSW Australia; 7 School of Life Sciences, Faculty of Science, University of Technology Sydney, Sydney, NSW Australia; 8 National Centre for Infectious Diseases, Singapore, Singapore; 9 Woolcock Institute of Medical Research, The University of Sydney, Sydney, NSW Australia; 10 Department of Respiratory and Sleep Medicine, John Hunter Hospital, New Lambton Heights, NSW Australia; 11 School of Chemical and Biomedical Engineering, Nanyang Technological University, Singapore, Singapore; 12 School of Biological Sciences, Nanyang Technological University, Singapore, Singapore; 13 Singhealth Duke-NUS Global Health Institute, Singapore, Singapore; 14 Department of Respiratory and Critical Care Medicine, Tan Tock Seng Hospital, Singapore, Singapore

**Keywords:** Chronic obstructive pulmonary disease, Infectious diseases, SARS-CoV-2

## Abstract

Chronic obstructive pulmonary disease (COPD) is characterised by airflow limitation and infective exacerbations, however, in-vitro model systems for the study of host-pathogen interaction at the individual level are lacking. Here, we describe the establishment of nasopharyngeal and bronchial organoids from healthy individuals and COPD that recapitulate disease at the individual level. In contrast to healthy organoids, goblet cell hyperplasia and reduced ciliary beat frequency were observed in COPD organoids, hallmark features of the disease. Single-cell transcriptomics uncovered evidence for altered cellular differentiation trajectories in COPD organoids. SARS-CoV-2 infection of COPD organoids revealed more productive replication in bronchi, the key site of infection in severe COVID-19. Viral and bacterial exposure of organoids induced greater pro-inflammatory responses in COPD organoids. In summary, we present an organoid model that recapitulates the in vivo physiological lung microenvironment at the individual level and is amenable to the study of host-pathogen interaction and emerging infectious disease.

## Abstract (web summary)

Chronic obstructive pulmonary disease (COPD) is a chronic inflammatory respiratory disease characterized by airflow limitation and infective exacerbations. Here, Chan et al. report the generation of nasopharyngeal and bronchial COPD organoids derived from adult stem cells and employ them in the study of host-pathogen interactions, including SARS-CoV-2 and Pseudomonas aeruginosa.

## Introduction

Chronic Obstructive Pulmonary Disease (COPD) is a chronic inflammatory respiratory disease of high global morbidity and mortality^1^. It is characterized by the development of progressive irreversible airflow limitation, heterogenous endophenotypes and differing disease trajectories between individual patients^2,3^. Patients suffer from persistent and progressive respiratory symptoms, impaired lung function, structural pulmonary abnormalities and exacerbations^4^. Patients with COPD also demonstrate a higher prevalence of developing co-morbidities such as cardiovascular disease that associate with poorer clinical outcomes and higher risks of mortality^5^. As the complexity and heterogeneity of COPD between individual patients is now widely recognized, cellular-based research remains highly challenging because of the lack of suitable experimental models that re-capitulate the disease at the individual level^6,7^. Small animal models including mice, guinea pigs and rabbits have all been used to study COPD, however, they cannot recapitulate the genetics and epigenetics of human disease, need COPD to be induced and once established does not necessarily mimic the entire clinical spectrum of observed endophenotypes in individual patients^8,9^. Therefore, establishing next generation cellular models that overcome such limitations and account for individual variation will be critical to better understand the molecular mechanisms that underpin infection and treatment responses at the individual level in COPD, permitting the application of bench-based precision medicine.

To facilitate such personalized studies, developing readily accessible in vitro models that reproduce the airway microenvironment in COPD at the individual level is paramount. To date, there remains a lack of suitable experimental models for the study of host-pathogen interaction in COPD. The past decade has witnessed major advances to the generation of stem cell-derived organoids, that incorporate multiple cell types within a 3-dimensional architecture^10^. Organoids represent a laboratory tool to recapitulate tissue-specific functional characteristics of their respective organ and facilitate the study of host-pathogen interaction. Human lung organoid models, reproducing the epithelial organization of their respective anatomical region, have been successfully generated from nasal^11,12^, bronchial^13^ and alveolar tissues^14,15^ using adult or pluripotent stem cells as starting materials^16–18^. Lung organoids have been used to study lung cancer^13,19^ and cystic fibrosis^13^ and to evaluate infection including respiratory syncytial virus (RSV)^13^, enterovirus^20^, cryptosporidium^21^, influenza^22,23^, and recently severe acute respiratory syndrome coronavirus 2 (SARS-CoV-2)^24–26^. Clinical and epidemiological data indicate that COPD associates with severe COVID-19 outcomes including a higher risk of hospitalization and death although driving mechanisms are lacking^27–34^.

Other than viral infection, bacterial infection accounts for a significant proportion of exacerbations in COPD, with the predominant species isolated including *Streptococcus pneumoniae* and *Pseudomonas aeruginosa*^35^.

Here, we show the establishment and characterization of COPD lung organoids and apply these to the study of host-pathogen interaction, including SARS-CoV-2 and *Pseudomonas aeruginosa*. Our COPD organoid models recapitulate the in vivo physiological lung microenvironment and result in elevated inflammatory responses to viral and bacterial infections.

## Results

### Establishment and characterization of human nasopharyngeal and bronchial organoids

**Fig. 1:**
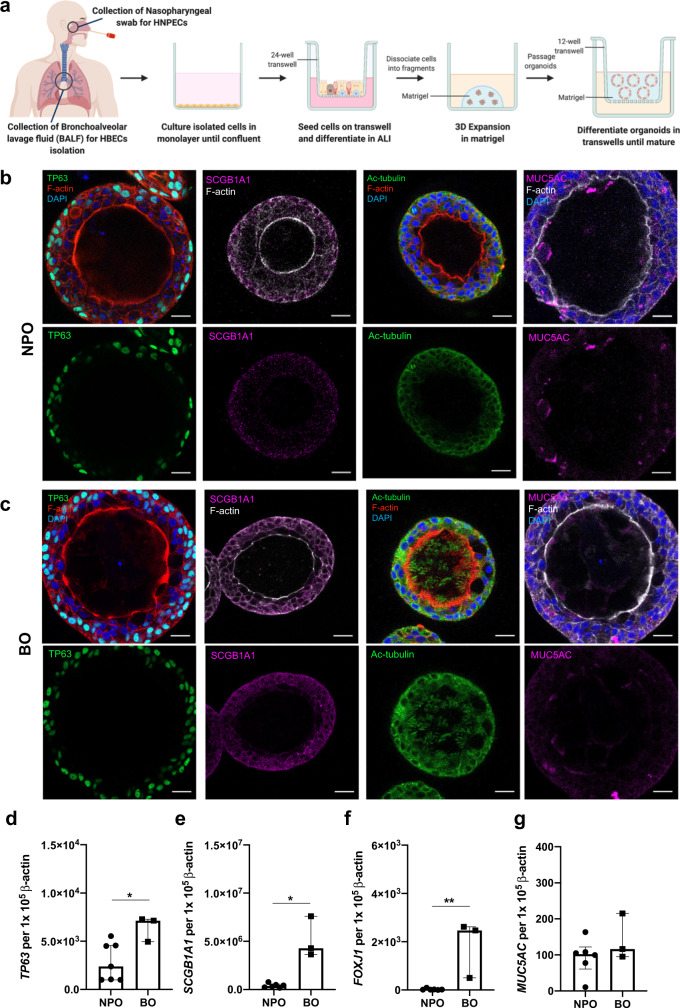
Establishment of nasopharyngeal and bronchial organoids from non-diseased individuals. **a** Schematic illustration of the isolation of primary human nasopharyngeal and bronchial epithelial cells and the generation of human organoids. **b** Immunofluorescence staining of human nasopharyngeal organoids (NPOs) (upper airway) for TP63 (basal cells: green), SCGB1A1 (club cells: purple), acetylated-α-tubulin (Ac-tubulin) (ciliated cells: green) and MUC5AC (goblet cells: purple). Nuclei and F-actin are counterstained with DAPI (blue) and Phalloidin (red/white), respectively. Scale bar = 20 μm. **c**. Immunofluorescence staining of human bronchial organoids (BOs) (lower airway). Scale bar = 20 μm. Data in b-c are representative of at least 5 independent experiments. **d–g** qRT-PCR analysis of total RNA extracted from NPOs and BOs for (D) *TP63* (basal cells), (E) *SCGB1A1* (club cells), (F) *FOXJ1* (ciliated cells) and (G) *MUC5AC* (goblet cells). *n* = 7 and *n* = 3 biologically independent experiments for NPOs and BOs, respectively, are illustrated. Data are presented as medians ± interquartile range and the Mann-Whitney U test is performed. *P < 0.05 [*TP63* = 0.0333; *SCGB1A1* = 0.0238; *FOXJ1* = 0.0238]. HNPEC: Human nasopharyngeal epithelial cells; BALF: Bronchoalveolar lavage fluid; HBEC: Human bronchial epithelial cells. Source data are provided as a Source Data file.

Primary human nasopharyngeal and bronchial organoids (NPOs and BOs, respectively) were established by methods previously reported with modification (Fig. 1a)^13^. A well-differentiated airway organoid develops a single or occasionally multiple lumen(s) lined by club, goblet, and ciliated cells. Basal cells are located exteriorly and motile cilia facilitating mucus swirling were visualized toward the interior (Fig. 1b, c, Supplementary Fig. 1 and Supplementary video 1). Immunofluorescence staining of non-diseased organoids demonstrated cellular markers for tumor protein 63 (TP63) + basal cells, secretoglobin Family 1 A Member 1 (SCGB1A1) + club cells, acetylated-α-tubulin (Ac-tubulin)+ ciliated cells and mucin 5AC (MUC5AC) + goblet cells in both NPOs and BOs (Fig. 1b, c, Supplementary Fig. 1). Marked differences were observed between NPOs and BOs, notably BOs were more ciliated with extensive Ac-tubulin+ cilia structures and higher FOXJ1 gene expression (Fig. 1b, c, Supplementary Fig. 1). Quantitative PCR (qPCR) confirmed that BOs express significantly higher *TP63, SCGB1A1* and *FOXJ1* compared with NPOs although, similar *MUC5AC* expression was detected between NPOs and BOs (Fig. 1d–g).

### Nasopharyngeal and bronchial organoids derived from COPD patients demonstrate higher MUC5AC gene expression and goblet cells

**Fig. 2:**
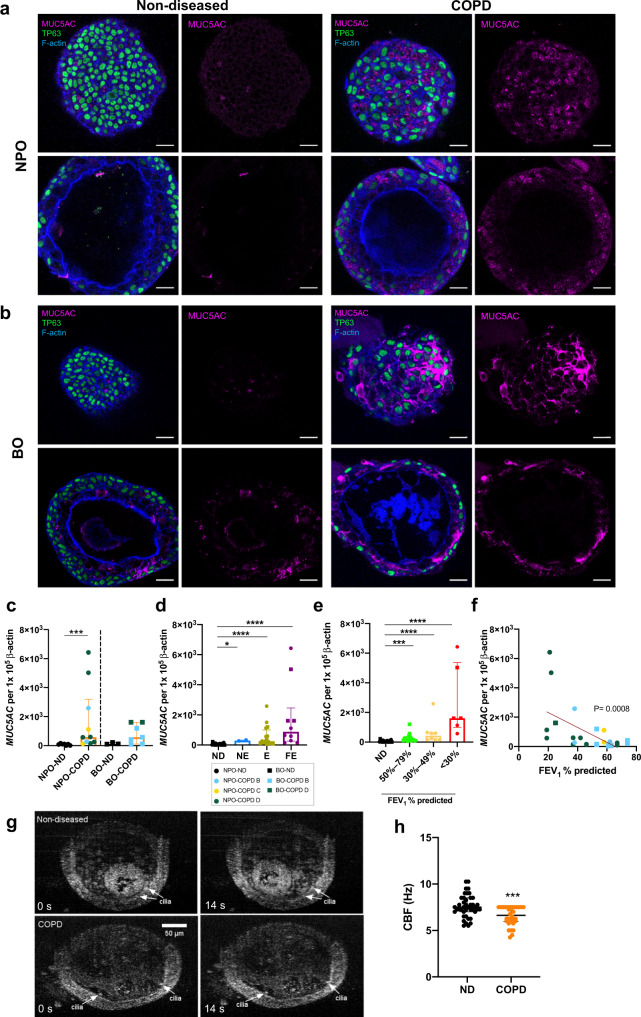
Nasopharyngeal and bronchial organoids from individuals with COPD demonstrate high MUC5AC expression and goblet cells. **a** Immunofluorescence staining of human nasopharyngeal organoids (NPOs) and **b** Human bronchial organoids (BOs) derived from non-diseased individuals (left) and patients with COPD (right) for MUC5AC (purple). The upper panel illustrates the organoid exterior while the lower panel illustrates the organoid lumen. Scale bar = 20μm. Data in a-b are representative of at least 5 independent experiments. **c** qRT-PCR analysis of total RNA extracted from NPOs and BOs derived from nondiseased individuals (ND) and COPD patients for *MUC5AC*. ND and COPD of GOLD stage B, C and D are denoted by black, blue, yellow, and green symbols, respectively. *n* = 7; *n* = 10; *n* = 3 and *n* = 8 biologically independent experiments for NPO-ND; NPO-COPD; BO-ND and BO-COPD are illustrated. Data are presented as medians ± interquartile range and the Mann-Whitney U test was performed. ***P < 0.001. [NPO-ND vs NPO-COPD = 0.0004] **d** *MUC5AC* gene expression in NPOs and BOs derived from ND and COPD, the latter stratified by exacerbation frequency as nonexacerbator (NE); exacerbator (E) and frequent-exacerbator (FE). *n* = 28 biologically independent samples are illustrated. Data are presented as medians ± interquartile range and the Mann-Whitney U test is performed. *P < 0.05; ****P < 0.0001. [ND vs NE = 0.0117; ND vs E < 0.0001; ND vs FE < 0.0001]. **e** Lung function as forced expiratory volume in the 1st second percent predicted (FEV_1_ % predicted). *n* = 28 biologically independent samples are illustrated. Data are presented as medians ± interquartile range and the Mann-Whitney U test was performed. ***P < 0.001; **** *P* < 0.0001. [ND vs 50–79%=0.0003; ND vs 30–49% <0.0001; ND vs <30% <0.0001]. **f** Scatter plot correlating MUC5AC gene expression in COPD organoids according to FEV_1_ % predicted. *n* = 28 biologically independent samples are illustrated. Spearman’s correlation coefficient (non-parametric) analysis was performed. *P* = 0.0008. **g** Representative images captured using micro-optical coherence tomography (μOCT) imaging of ND and COPD bronchial organoids at 0 and 14 s (s). Scale bar = 50 μm. Data in g are representative of 3 independent experiments. **h** Quantification of ciliary beat frequency (CBF) measurement in Hertz (Hz) of bronchial organoids derived from ND (*n* = 3) and COPD (*n* = 3). 6 to 10 organoids are evaluated per donor in up to 10 regions of ciliary activity per image sequence to determine the CBF. Data are presented as medians ± interquartile range and the Mann-Whitney U test is performed. ***P < 0.001 [*P* = 0.0008]. Source data are provided as a Source Data file.

NPOs and BOs derived from COPD subjects (GOLD stage B, C and D) contain all the cell types observed in non-diseased organoids (Fig. 2a, b, Supplementary Fig. 1). A greater abundance of goblet cells was characteristic of COPD organoids, including significantly elevated *MUC5AC* gene expression in COPD compared to non-diseased NPOs (Fig. 2a–c). In COPD BOs, diminished club and ciliated cell populations were evident, characteristic of the disease state (Supplementary Fig. 1, 2b, c). When COPD organoids were stratified by exacerbations in their donors, an increasing exacerbation frequency significantly related to a higher *MUC5AC* gene expression in their respective organoid culture (Fig. 2d). A close relationship is observed with pulmonary function, where those with the poorest lung function (<30% FEV_1_ % predicted) demonstrate the highest *MUC5AC* gene expression, further exemplified by a significant inverse relationship between *MUC5AC* gene expression and FEV_1_ % predicted (*R* = − 0.5890; *p* = 0.0008; Fig. 2e, f). Importantly, this cellular association to clinical COPD correlates does not extend to other cell types present in the organoid model (Supplementary Fig. 2). COPD exhibits an impaired mucociliary clearance that predisposes to infection, and the presence of such dysfunction was assessed in the derived non-diseased and COPD BOs by micro-optical coherence tomography (µOCT) imaging. BOs demonstrated a clear visualization of functional cilia, and ciliary beat frequency (CBF) was significantly lower in COPD BOs when compared to non-diseased (Fig. 2g, h and Supplementary video 2).

### Single-cell characterization of non-diseased and COPD lung organoids

**Fig. 3:**
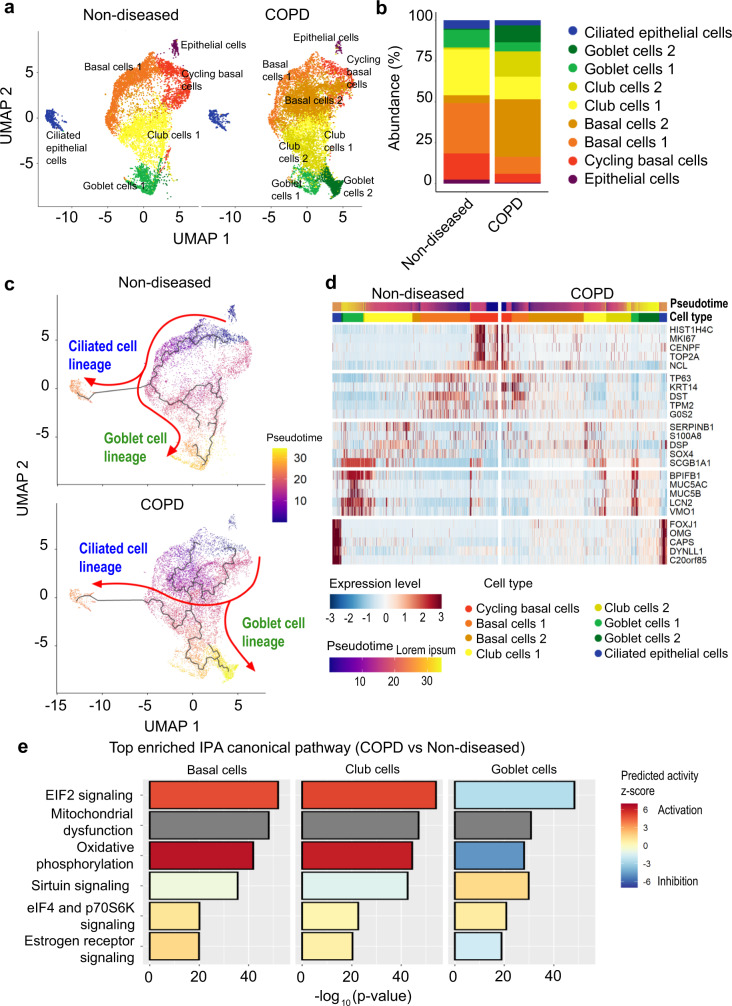
Single cell RNA-seq reveals developmental and functional cellular impairment in COPD organoids. **a** UMAP plots of scRNA-seq transcriptomic data highlight the main cell types detected in lung organoids of non-diseased (ND) (left; *n* = 8614 cells) (pooling of one NPO-ND line and one BO-ND line) and COPD (right; *n* = 11,263 cells) (pooling of one NPO-COPD line and one BO-COPD line). **b** Stacked bar plots of the relative abundance for the nine main cell types in ND and COPD organoids. **c** Pseudotime analysis of ND (top) and COPD organoids (bottom) illustrating the variable trajectory of cycling basal cells into ciliated and goblet cell lineages. **d** Heatmap illustrating altered expression pattern, based on cell type-specific markers, in ND and COPD organoids. Cells are ordered by their respective disease state: ND or COPD, cell type, pseudotime and illustrated by expression level by the colour keys provided. **e** Bar graphs illustrating the top enriched Ingenuity Pathway Analysis (IPA) canonical pathways derived from differentially expressed genes of COPD to ND organoids for basal, club and goblet cells respectively. The predicted activity z-score from IPA is colour-coded (red: activation; blue: inhibition; grey: no activity pattern available).

Single-cell transcriptomes demonstrate that our in vitro lung organoids faithfully recapitulate major cell populations found in the native in vivo airway, including cycling basal cells, basal cells, club cells, goblet cells, and ciliated epithelial cells (Fig. 3a, Supplementary Figure 3a) in both NPOs and BOs (Supplementary Fig. 3c, d). Although in a small sample size, this result is in line with our immunofluorescence and qPCR analysis as illustrated (Figs. 1, 2). Cell identities were annotated based on the airway and lung cell signature gene sets derived from Garcia et al. (2019) and Travaglini et al. (2020) using marker genes for each respective cell cluster^36,37^ (Supplementary Data 1). We further confirmed cell identities using specific marker genes, for instance, *MKI67* for cycling basal cells, *TP63* and *KRT5* for basal cells, *SCGB1A1* for club cells, *MUC5AC* for goblet cells, and *FOXJ1* for ciliated epithelial cells (Supplementary Fig. 3b). A single minor cluster comprising ~1.4% of the total cells was detected, demonstrating only *KRT5* expression, and hence we conservatively classed these as “epithelial cells”. When compared to non-diseased organoids, COPD organoids demonstrated fewer cycling basal (5.4% versus 16.0%) and ciliated epithelial cells (3.2% versus 5.2%) but more basal (45.7% versus 35.6%) and goblet cells (15.9% versus 11.5%). Club cell populations were comparable (29.4% versus 29.3%) (Fig. 3b). The higher abundance of goblet cells, and lower proportion of ciliated cells are indicative of the goblet cell hyperplasia and ciliary abnormalities characteristic of COPD.

Next, we assessed publicly available bulk RNAseq datasets derived from the small airways in COPD (GSE162154) and compared the expression profile of DEGs to those observed in the pseudobulk analysis of our scRNAseq data. Hierarchical clustering reveals distinct clusters of healthy and COPD samples. Key functional pathways detected in COPD organoids, including mitochondrial dysfunction, endoplasmic reticulum stress, cilium reorganization, extracellular matrix remodelling and proinflammatory cytokine signalling were common to both the pseudobulk analysis and public datasets (Supplementary Fig. 4b, c). Basal cells from COPD organoids demonstrate a highly consistent functional pattern not observed in other cell types and exhibit an altered developmental trajectory of cycling basal cells into ciliated and goblet cell lineages (Fig. 3c). High abundance of basal cells in the airway and in organoids may result in the under-representation of other cell types in bulk RNAseq analysis. This is reflected by the functional similarities observed between basal cell transcriptomes from lung organoids and the publicly accessible bulk RNAseq COPD datasets (Supplementary Fig. 4c).

Capturing transcriptomes at single-cell resolution in COPD organoids allows assessment of functional change associated with minor cell types, unappreciable by bulk RNAseq. Two sub-populations each of basal, club and goblet cells were identified with significantly different abundance between non-diseased and COPD organoids, where COPD organoids are over-represented by the following groups: Basal cells 2 (35.3% versus 4.7%), Club cells 2 (15.6% versus 1.2%) and Goblet cells 2 (10.4% versus. 0.7%), none of which are observed in non-diseased organoids (Fig. 3b–d). The Basal cells 2 and Club cells 2 groups also represent the predominant intermediate cell type observed in the altered developmental trajectory of basal cells in COPD organoids (Fig. 3b–d). COPD organoids demonstrate an impaired transition from one cell type to another when compared to non-diseased organoids (Fig. 3c, d). This is evidenced by a clear loss of marker gene expression in Basal cells 2, Club cells 2, and Goblet cells 2, all over-represented in COPD organoids and their detectable expression of marker genes from other cell types (Fig. 3b–d, Supplementary Figs. 3d, 4a). Taken together, this suggests a punctuated differentiation of cells from COPD organoids, likely attributable to COPD pathogenesis.

To further interrogate functional pathways differing between non-diseased and COPD organoids, we assessed functional differences between the basal, club and goblet cells of each organoid model. All three cell types in COPD organoids demonstrate perturbation of similar canonical pathways (Fig. 3e). In basal and club cells, the EIF2 pathway and oxidative phosphorylation were markedly activated while sirtuin signalling was mildly suppressed. Inverse trends were observed in goblet cells. Independently, gene-set enrichment analyses (GSEA) captured similar derangements but additionally uncovered immune cell activation in all three airway cell types (Supplementary Fig. 4d).

### Lung organoids are permissive to SARS-CoV-2 infection

**Fig. 4:**
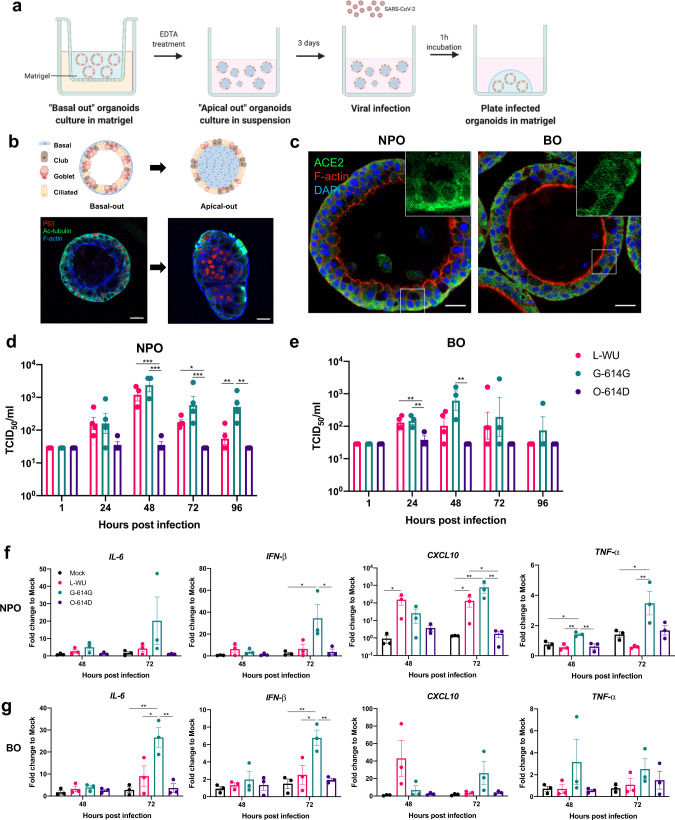
Lung organoids are permissive to SARS-CoV-2 infection. **a** Schematic diagram outlining the methodological workflow for SARS-CoV-2 infection of NPOs and BOs. **b** Pictorial representation of ‘basal-out’ and ‘apical-out’ airway organoids (top) and immunofluorescence staining of NPOs in ‘basal-out’ and ‘apical-out’ polarity for TP63 (basal cells: red) and Ac-tubulin (ciliated cells: green) (bottom). Scale bar = 20 μm. **c** Immunofluorescence staining of NPOs and BOs derived from non-diseased individuals for ACE2 (green). Scale bar = 20 μm. Data in b-c are representative of at least 5 independent experiments. **d–e** Viral replication kinetics from culture supernatants harvested from SARS-CoV-2-infected (**d**) NPOs and (**e**) BOs at 1, 24, 48, 72 and 96 h post infection (hpi) by TCID_50_ assay. *n* = 4 and n = 3 biologically independent experiments for NPOs and BOs, respectively. Data are presented as means ± SEM and one-way ANOVA is performed. *P < 0.05; **P < 0.01; ***P < 0.001 [NPO: 48 hpi: L-WU vs O-614D = 0.0005; G-614G vs O-614D = 0.0002; 72 hpi: L-WU vs O-614D = 0.0170; G-614G vs O-614D = 0.0006; 96 hpi: L-WU vs G-614G = 0.0041; G-614G Vs O-614D = 0.0011; BO: 24 hpi: L-WU vs O-614D = 0.0070; G-614G vs O-614D = 0.0065; 48 hpi: G-614G vs O-614D = 0.0048]. **f–g** qRT-PCR of total RNA extracted from SARS-CoV-2 infected (**f)** NPOs and (**g**) BOs respectively at 48 and 72 hpi for interleukin-6 (*IL-6*), interferon-β (*IFN-β*), C-X-C motif chemokine ligand 10 (*CXCL10*) and tumour necrosis factor-α (*ΤΝF-α*). *n* = 3 biologically independent experiments are illustrated. Data are presented as means ± SEM and one-way ANOVA is performed. *P < 0.05; **P < 0.01. [NPO: *IFN-β:* MK vs G-614G = 0.0399; G-614G vs O-614D = 0.0484; *CXCL10:* 48hpi: MK vs L-WU = 0.0156; 72hpi: MK vs L-WU = 0.0259; MK vs G-614G = 0.0018; G-614G vs O-614D = 0.0017; L-WU vs O-614D = 0.0236; *ΤΝF-α:* 48hpi: MK vs G-614G = 0.0254;L-WU vs G-614G = 0.0069; G-614G vs O-614D = 0.0098; 72hpi: MK vs G-614G = 0.0374;L-WU vs G-614G = 0.0062] [BO: *IL-6*: MK vs G-614G = 0.0054; L-WU vs G-614G = 0.0282; G-614G vs O-614D = 0.0066; *IFN-β:* MK vs G-614G = 0.0055; L-WU vs G-614G = 0.0185; G-614G vs O-614D = 0.0092]L-WU: Clade L-Wuhan strain; G-614G: Clade G strain and O-614D: Clade O strain. Source data are provided as a Source Data file.

Our derived human lung organoids are amenable for the study of host-pathogen interaction. To facilitate such infection studies and permit direct access of the infecting microorganism to the luminal cell population, NPOs and BOs were re-orientated to achieve apical-out polarity (Fig. 4a, b). Organoids with outwardly oriented cilia were differentiated within 72 h in suspension and basal cells positioned in the organoid interior without a visible lumen. Functionally competent cilia, now outwardly oriented, induce self-rotation of the entire organoid structure (Supplementary video 3).

NPOs and BOs from non-diseased individuals and COPD express *ACE2*, *TMPRSS2*, *furin* and *neuropilin-1*, all of which play key roles in SARS-CoV-2 infection (Supplementary Fig. 5a–d). Of note, *ACE2*, *TMPRSS2* and *neuropilin-1* gene expression were significantly higher in the lower airway in non-diseased donors with no differences between the upper and lower airways in COPD (Supplementary Fig. 5a–d). *ACE2* was detected in NPOs and BOs extraneous to their polarity and unrelated to cell type or distribution (Fig. 4c). Our single cell analysis reveals that *ACE2* is expressed in several cell types, and *TMPRSS2* was pervasive in most cell types, especially ciliated epithelial cells, while *furin* largely colocalized with *neuropilin-1* (Supplementary Fig. 6). Interestingly, COPD-NPOs exhibited higher *ACE2* and *TMPRSS2* expression compared to non-diseased individuals, whilst a contrasting pattern is observed in COPD-BOs (Supplementary Figs. 5a,b, 6).

Significant relationships are detected between increasing COPD exacerbation frequency and higher gene expression for all four SARS-CoV-2 factors (Supplementary Fig. 5e–h). When lung function is considered, a significantly higher expression of *TMPRSS2*, *furin* and *neuropilin-1* are observed in COPD with the poorest lung function (<30% FEV_1_ % predicted) and, only *furin* demonstrated a significant inverse relationship with FEV_1_ % predicted (*R* = − 0.4880; *p* = 0.0072) (Supplementary Fig. 5i–p).

Spontaneous mutations in the SARS-CoV-2 genome, acquired continuously throughout the pandemic, alters viral fitness. We, therefore, selected three patient isolates to evaluate their differential pathogenesis: an ancestral Wuhan strain in Clade L (L-WU, GISAID accession ID: EPI_ISL_407987), a Clade O strain (O-614D, GISAID accession ID: EPI_ISL_574486), and a Clade G strain (G-614G, GISAID accession ID: EPI_ISL_574489). Amino acid changes were identified in the spike protein-D614G, ORF1ab, and a stop codon detected in NS6 between G-614G and L-WU while, more mutations in ORF1ab, S, NS7a, and N were detected in O-614D (Supplementary Table 1). SARS-CoV-2 infection did not induce cytopathic effects in NPOs over the infection period of 96 h (Supplementary Fig. 7). Viral infectivity, assessed by qPCR measured at 48 and 72 h post-infection (hpi) demonstrated no significant differences between the three isolates or between the upper and lower airway (Supplementary Fig. 8a–c). L-WU and G-614G productively replicated in NPOs and BOs while O-614D failed to replicate despite demonstrable productive replication in Vero-E6 cells (Fig. 4d, e, Supplementary Figs. 8d–f and 10). In NPOs, representative of upper airways and an important site for SARS-CoV-2 transmissibility, the G-614G replicated to significantly higher titers compared to L-WU at 96 hpi and extended to 168 hpi (Fig. 4d, Supplementary Fig. 8g). Additionally, G-614G infected organoids elicit enhanced proinflammatory immune responses at 72 hpi characterized by enhanced interferon-β (*IFN-β*), C-X-C motif chemokine ligand 10 (*CXCL10*), and tumour necrosis factor-α (*ΤΝF-α*) gene expression in NPOs and interleukin-6 (*IL-6*) and *IFN-β* in BOs, respectively (Fig. 4f, g). G-614G infection of NPOs and BOs significantly increases CXCL10 at 72 hpi (Supplementary Figs. 8h and 9).

### SARS-CoV-2 infection exhibits higher replication competence in COPD bronchi

**Fig. 5:**
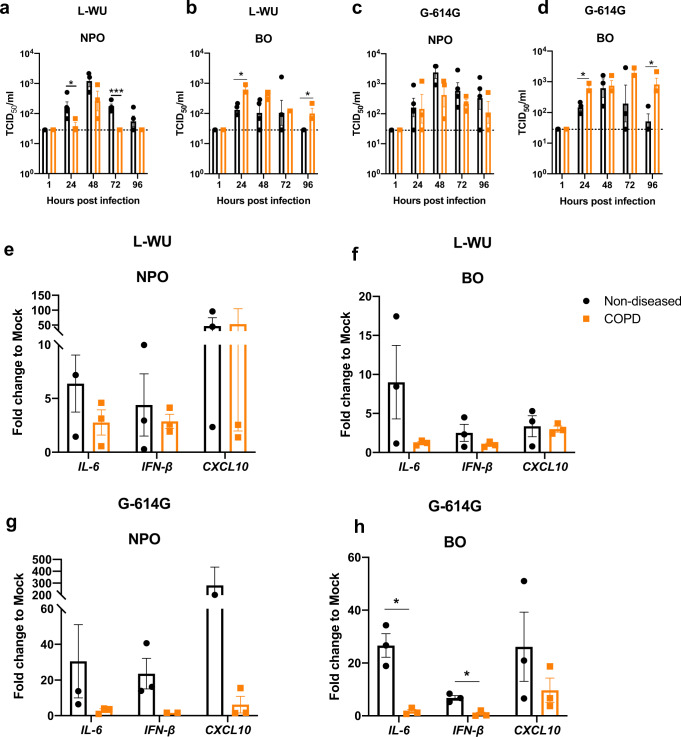
SARS-CoV-2 infection exhibits higher replicative competence in COPD bronchial organoids. **a–d** Viral replication kinetics from L-WU-infected and G-614G infected NPOs and BOs at 1, 24, 48, 72 and 96 hpi by TCID_50_ assay. *n* = 3 biologically independent experiments for NPO and BO infections are illustrated. Data are presented as means ± SEM and unpaired t-test is performed. *P < 0.05; ***P < 0.001. [NPO L-WU: 24 hpi=0.0494; 72 hpi=0.0004; BO L-WU: 24 hpi=0.0110; 96 hpi=0.0152; BO G-614G: 24 hpi=0.0294; 96 hpi=0.0457]. **e–h** qRT-PCR analysis of total RNA extracted from L-WU-infected and G-614G -infected NPOs and BOs derived from non-diseased individuals and COPD patients respectively at 72 hpi for *IL-6, IFN-β* and *CXCL10*. *n* = 3 biologically independent experiments are illustrated. Data are presented as means ± SEM and an unpaired t-test is performed. *P < 0.05. [Fig. 5h: *IL-6* = 0.0237*; IFN-β* = 0.0248] Source data are provided as a Source Data file.

Clinical and epidemiological data propose COPD as an independent risk factor for severe COVID-19^34^. We therefore next assessed infectivity, viral replication, proinflammatory and antiviral responses in airway organoids to elucidate cellular correlates for these clinical findings. Investigations were based on infection with the L-WU and G-614G strains but not O-614D because the latter failed to replicate and induce inflammatory responses in the non-diseased models. High levels of SARS-CoV-2 infectivity, comparable to that in the non-diseased state, were detected in COPD organoids from the upper and lower airway at 48 and 72 hpi (Supplementary Fig. 11). Interestingly, viral replication kinetics reveal that L-WU replicated less efficiently in NPOs, however enhanced replication was observed in the BOs derived from COPD patients (Fig. 5a, b). No differences for the G-614G variant were observed between COPD and non-diseased NPOs, although a significantly enhanced replication in the lower airways (i.e. BOs) was again evident in the former (Fig. 5c, d). Of note, cell lysates (detecting nucleocapsid (NS) gene expression) and culture supernatants (detecting live infectious virus) were used in the qPCR and TCID_50_ experiments respectively accounting for observed differences between measured viral load and viral titres. Taken together however, this suggests an overall enhanced replication competence of the G-614G, and critically, a significantly increased and robust potential for viral replication in COPD bronchi, the predominant site for the development of severe COVID-19^34^ (Fig. 5c, d). qPCR assessment of *IL-6, IFN-β* and *CXCL10* revealed no differences between the NPOs and BOs following L-WU infection with and without COPD. However, infection with G-614G demonstrated a significant reduction in *IL-6* and *IFN-β* in the lower COPD airway with a trend toward inhibition of *CXCL10* when compared to non-diseased individuals (Fig. 5e–h).

### COPD NPOs demonstrate enhanced pro-inflammatory responses to *Pseudomonas aeruginosa* and *Streptococcus pneumoniae* infection

**Fig. 6:**
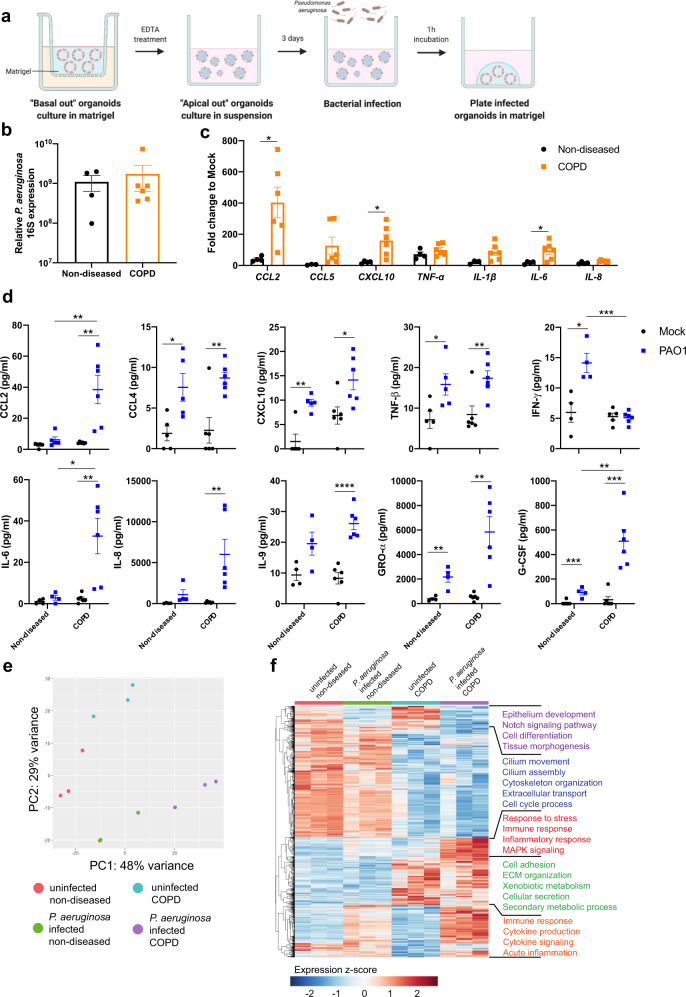
Nasopharyngeal organoids are permissive to bacterial infection by *Pseudomonas aeruginosa*. **a** Schematic diagram outlining the method for bacterial infection of NPOs. **b** qRT-PCR analysis of total RNA extracted from *P. aeruginosa*-infected NPOs derived from non-diseased individuals and COPD patients at 6 hpi for the *P. aeruginosa* 16 S gene. *n* = 4 and *n* = 6 biologically independent experiments were performed for ND and COPD, respectively. Data are presented as means ± SEM. **c** qRT-PCR analysis of *P. aeruginosa*-infected NPOs at 6 hpi for C–C motif chemokine ligand 2 (*CCL2*), *CCL5*, C-X-C motif chemokine ligand 10 (*CXCL10*), tumour necrosis factor-α (*ΤΝF-α*), interleukin-1β (*IL-1β*), *IL-6* and *IL-8*. *n* = 4 for ND and *n* = 6 for COPD. Data are presented as means ± SEM and an unpaired t-test is performed. *P < 0.05 [*CCL2* = 0.0188; *CXCL10* = 0.0239; *IL-6* = 0.0470]. **d** CCL2, CCL4, CXCL10, TNF-β, interferon-γ (IFN-γ), IL-6, IL-8, IL-9, growth-related oncogene- α (GRO-α) and granulocyte-colony stimulating factor (G-CSF) release upon *P. aeruginosa* infection in NPOs at 6 hpi by multiplex Luminex assay. *n* = 5 for ND and *n* = 6 for COPD. Data are presented as means ± SEM and an unpaired t-test is performed. *P < 0.05; **P < 0.01; ***P < 0.001; **** *P* < 0.0001. [CCL2: COPD-MK vs COPD-PAO1 = 0.0039; ND-PAO1 vs COPD-PAO1 = 0.0062, CCL4: ND-MK vs ND-PAO1 = 0.0190; COPD-MK vs COPD-PAO1 = 0.0040; CXCL10: ND-PAO1 = 0.0014; COPD-MK vs COPD-PAO1 = 0.0217; TNF-β: ND-MK vs ND-PAO1 = 0.0333; COPD-MK vs COPD-PAO1 = 0.0096; IFN-γ: ND-MK vs ND-PAO = 0.0114; ND-PAO1 vs COPD-PAO1 = 0.0002; IL-6: ND-PAO1 vs COPD-PAO1 = 0.0238; COPD-MK vs COPD-PAO1 = 0.0055; IL-8: COPD-MK vs COPD-PAO1 = 0.0099; IL-9: COPD-MK vs COPD-PAO1 < 0.0001; GRO-α: ND-MK vs ND-PAO1 = 0.0062; COPD-MK vs COPD-PAO1 = 0.0018; G-CSF: ND-MK vs ND-PAO1 = 0.0004; COPD-MK vs COPD-PAO1 = 0.0005; ND = PAO1 vs COPD-PAO1 = 0.0067]. **e** Principal component analysis (PCA) of ND and COPD NPO transcriptomes that were mock-treated or infected with *Pseudomonas aeruginosa* for 6 h (*n* = 3 per group). **f** Heatmap of the differentially expressed genes (2561 genes) between *P. aeruginosa* infected and uninfected NPOs from ND and COPD, respectively. Five major gene clusters based on expression profile were identified and biological functions are annotated. Source data are provided as a Source Data file.

The clinical course of COPD is punctuated by episodes of clinical deterioration termed exacerbations. Bacterial infections account for a proportion of such exacerbations, and, here we investigated the inflammatory outcomes from *Pseudomonas aeruginosa* and *Streptococcus pneumoniae* using our “apical-out” NPOs from COPD patients (Fig. 6 and Supplementary Fig. 11). Nondiseased and COPD NPOs were comparably susceptible to infection by either bacterium, and COPD NPOs induced a greater proinflammatory response to bacterial infection at RNA and protein levels, demonstrating the value of our COPD organoid model in assessing the host response to infection (Fig. 6b–d and Supplementary Fig. 11). Bulk transcriptomic profiling of *P. aeruginosa*- infected COPD NPOs demonstrate significantly impaired ciliary movement and increased secretory and extracellular matrix remodelling activities in the uninfected state when compared to non-diseased NPOs. As expected, *P. aeruginosa* infection of non-diseased NPOs triggered acute inflammation with elevated cytokine production and signalling (Fig. 6e, f). Notably, inflammatory and stress responses were significantly enhanced in infected COPD NPOs.

## Discussion

Here, we demonstrate the establishment and characterization of COPD organoids, derived from adult stem cells to study host-pathogen interaction. COPD organoids are multicellular 3-dimensional spheroid structures, exhibiting essential cellular characteristics of COPD. COPD organoids demonstrate goblet cell hyperplasia and reduction in ciliary beat frequency, related to the underlying disease severity of their respective donors and, representative of the expected disease pathology. Single-cell analysis of COPD organoids revealed developmental and functional abnormalities representative of the disease state. SARS-CoV-2 infection of COPD organoids reveals more productive replication in bronchi when compared to healthy individuals providing a potential basis for the clinical observations of poorer COVID-19 outcomes in COPD.

Airway remodelling, in response to chronic, recurrent airway injury and inflammation is an important pathological feature of chronic respiratory disease, including COPD^38–40^. In addition, mucus hypersecretion and a high abundance of goblet cells characterize the COPD airway promoting airway obstruction, impaired mucociliary clearance, and exacerbations^41–44^. COPD organoids recapitulate this goblet cell hyperplasia phenotype and exhibit higher *MUC5AC* gene expression and reduced ciliary beat frequency in comparison to healthy individuals. Interestingly, *MUC5AC* gene expression in the organoids is significantly associated with a COPD donor’s underlying lung function, disease severity, and exacerbation frequency, and a significant inverse relationship between *MUC5AC* gene expression and lung function was apparent. This negative correlation is in line with a recent multicentre study performed on the COPD SPIROMICS cohort demonstrating increased MUC5AC sputum concentrations in COPD in association with poorer lung function (FEV_1_ % predicted)^45^. MUC5AC has therefore been proposed as a potential biomarker to prognosticate COPD.

Single-cell transcriptomes of COPD organoids, in the absence of exposure to cigarette smoke, inflammatory stimuli, proteolytic enzymes, or genetic modification, reveals a high consistency with existing in vitro and in vivo COPD models at the phenotypic, transcriptomic, and functional levels^46,47^, albeit in a small sample size. These analyses also further reveal the COPD goblet cell hyperplasia and illustrate a punctuated cellular differentiation in COPD due to the inherently abnormal cellular development that characterizes its pathogenesis. Our pseudotime analysis is consistent with existing studies that demonstrate defects in tissue development, regeneration, and epithelial differentiation, including NOTCH, Wnt/β-catenin, and Hedgehog signalling pathways observed in the COPD airway^48–50^. Biological pathways associated with COPD, including mitochondrial dysfunction and sirtuin signalling were uncovered by our single-cell characterization of COPD organoids, which offer the significant advantage of assessing minor cell populations not reflected in bulk RNAseq^51–53^. Disturbance to mitochondrial structure and function in lung epithelia influences key processes ranging from cellular differentiation, cell death, and cellular remodelling to physical barrier functions and innate immunity, all of which are linked to COPD pathogenesis through cigarette smoke exposure^54^. Dysregulated mitochondrial biogenesis together with an increased production of reactive oxygen species (ROS) may cause irreversible cell growth arrest or senescence, especially when accompanied by an imbalance between oxidant-antioxidant capacity, as seen in COPD^55,56^. In line with our findings, this is most evident in club cells, cells highly sensitive to oxidative damage compared to other airway epithelial cell types^57^. In addition, sirtuin 1 (SIRT1) has been implicated in the development and progression of COPD and in the susceptibility to viral infection^58,59^. Decreased levels of SIRT1 are observed in COPD lungs and associate with enhanced inflammation by increasing acetylation of nuclear RelA/p65 and IL-8 release^59,60^. Consistently, increased activation of genes associated with the immune response is detectable in basal, club and goblet cells from COPD organoids. Interestingly, distinct cellular profiles were identified in COPD BOs but not other organoids where “Basal cells 2”, “Club cells 2” and “Goblet cells 2” were over-represented. The nasal epithelium is suggested to act as a proxy for the bronchial region in the detection of COPD-associated genes^61,62^, however, we demonstrate that our organoid models derived from the upper and lower airways display similar COPD-associated gene expression profiles, but different cellular profiles.

Current evidence suggests that individuals with COPD are at higher risk for severe COVID-19, including hospitalization, ICU admission, need for mechanical ventilation, and mortality^27,28,63–66^. In this study, our organoid models, regardless of clinical background, demonstrate expression of the key SARS-CoV-2 entry factors and their related endogenous proteases, including *ACE2*, *TMPRSS2*, *furin*, and *neuropilin-1*^67–72^. *ACE2* and *TMPRSS2* expression was significantly elevated in COPD organoids (NPOs), findings in line with existing reports seen in airways of smokers and COPD patients^73–76^. Using three phylogenetically distinct SARS-CoV-2 clades identified in 2020, namely clade L, clade O and clade G, we illustrate that the G clade isolate containing a D614G spike mutation replicated to higher viral titres and demonstrated more robust proinflammatory responses^77,78^, even at extended timepoints, in comparison to clade L and O strains lacking a D614G mutation. This is in line with prior work in animal^79–82^ and human airway models^81,83^. Critically, we demonstrate that COPD organoids were more vulnerable to SARS-CoV-2 infection resulting in greater replication and inhibition in *IFN-β* expression, specifically in the bronchial region, following infection with G-614G. This higher replication competence, coupled with an impaired antiviral immune response following G-614G infection, provides potential mechanistic insight to in part explain the worse clinical outcomes observed in COPD patients with COVID-19. Intriguingly, more efficient viral replication was only demonstrated in BOs derived from COPD patients, while, a contrasting effect was observed in NPOs in comparison to non-diseased counterparts. This interesting result may possibly be contributed to by the over-representation of the cell populations of “Basal cells 2”, “Club cells 2” and “Goblet cells 2” in COPD BOs coupled to inhibition of *IFN-β*. Consistent with our findings, a recent study has demonstrated that bronchial epithelial cells derived from COPD patients are more susceptible to SARS-CoV-2 infection because of their enrichment for co-receptor expression, protease imbalances, and stronger inflammatory responses^84^.

Viruses remain a key trigger for COPD exacerbations^85^, and deficient antiviral immunity in COPD associates with a greater risk for virus-induced exacerbation^86,87^. Reduced IFN expression in COPD bronchi following rhinovirus and/or influenza infection is observed, while resected lungs from stable COPD demonstrate a constitutive impairment of IFN-β, IRF-7 and other interferon- stimulated genes (ISGs)^88–91^. Frequent COPD exacerbators, most marked in those with severe disease, display further depletion of type I and III interferons and other ISGs in sputum even during clinical stability^91–93^. Importantly, this work further augments these observations by illustrating that impairment of the interferon response may be similarly relevant following SARS-CoV-2 infection.

Bacteria similarly have important roles as causative agents in COPD exacerbations where *Streptococcus pneumoniae* and *Pseudomonas aeruginosa* represent two of the most frequently isolated bacterial species^94–96^. Bacteria are isolated from COPD sputum in up to 50% of those with moderate to severe disease during stability, which increases to two-thirds of individuals at exacerbation^97,98^. Inflammatory consequences from the presence of bacteria in the COPD airway, including those associated with acute exacerbations, are associated with poorer lung function and prolonged hospitalization^99,100^. COPD organoids (NPOs), particularly those derived from frequent exacerbators, reveal enhanced inflammatory responses to both *S. pneumoniae* and *P. aeruginosa* despite comparable permissibility to infection, contributing to poorer clinical outcomes observed in this patient group. The use of COPD organoids potentially allows insight into the inflammatory response, at the individual level, to potentially pathogenic organisms and may be useful in applying precision approaches to the assessment of anti-inflammatory or anti-microbial therapies in COPD.

While we establish and characterize COPD organoids for the first time, our study does have several limitations. Nasopharyngeal and bronchial sampling for use as starting material to generate organoid models were collected from different individual donors rather than paired specimens from an individual. Human samples used to generate non-diseased (healthy) organoids came from relatively younger donors compared to COPD specimens. Our presented scRNA analysis was based on a single sample from each respective group, and therefore further experimentation is warranted to validate these findings. While our COPD organoids are physiologically relevant and representative of the human airway epithelium, they lack complexity such as the immune and vasculature systems, an ongoing challenge in the field. While it would have been of interest to obtain scRNA data from SARS-CoV-2 infected organoids, these experiments were precluded on grounds of safety and logistics.

In summary, we establish and characterize COPD organoids to study host-pathogen interaction that structurally and functionally represents the underlying disease state. Such models allow assessment of the response to infection and/or drug treatment, at the individual level, and may form a useful addition in future pandemic preparedness strategies where they are amenable to high throughput therapeutic screening and disease-based assessment of evolving pathogens.

## Methods

### Subject recruitment

Twenty-eight individuals were recruited for the cultivation and characterization of NPOs and BOs, including non-COPD (healthy) volunteers (*n* = 10) and individuals with COPD (*n* = 18). Flocked nasopharyngeal swabs (FNPS) and/or bronchial specimens, collected through lung resection and/or fibre-optic bronchoscopy as described below, were obtained as starting material for experimentation.

### Non-COPD (healthy) individuals

Subjects with normal spirometry and no prior history of COPD or any other respiratory disease were recruited. All participants were lifelong non-smokers (except for a single ex-smoker) and all participants were not on any long-term medication. Participant demographics are summarised in Supplementary Table 2.

### COPD

Patients aged ⩾45 years with stable COPD attending respiratory outpatient clinics at tertiary referral centres for routine follow-up were recruited at three hospitals across two countries as follows: Singapore General Hospital (Singapore), St Vincents Hospital (Sydney, Australia) and the John Hunter Hospital (Newcastle, Australia). COPD was defined according to the Global Initiative for Chronic Obstructive Lung Disease (GOLD) criteria^1,101^. Patients with any prior history of asthma (defined by variable symptoms and expiratory airflow limitation according to the Global Initiative for Asthma guidelines; www.ginasthma.org) and those receiving long-term oral steroids, or any immunosuppressive agents were excluded. Stable COPD was defined as the absence of an exacerbation four weeks prior to study recruitment. Non-exacerbators (NE) were defined by the absence of any documented exacerbation in the year preceding study recruitment, while COPD exacerbators (E) and frequent exacerbators (FE) were defined as having less than or more than two exacerbations, respectively in the year preceding study recruitment. A COPD exacerbation was defined as sudden deterioration of respiratory symptoms (cough, sputum production, shortness of breath and/or wheeze) requiring additional therapy (steroids and/or antibiotics) as determined by the patient’s primary respiratory physician^1^. For frequent COPD exacerbators, a prior history of recurrent exacerbations despite receiving COPD therapy based on GOLD guidelines was documented^1,101^. All recruited patients were receiving appropriate COPD therapy (including smoking cessation counselling, inhaler assessment, COPD action plans, inhalers as long-acting β-agonists, long-acting muscarinic antagonists, inhaled corticosteroids and/or short-acting bronchodilators in addition to vaccination as appropriate) based on GOLD guidelines^1,101^. All patients underwent full spirometry (to determine forced expiratory volume in the 1st second percent predicted (FEV1 % predicted).; FEV_1_ forced vital capacity; FVC and FEV_1_/FVC ratio) in line with the technical standards defined by ATS/ERS criteria followed by nasopharyngeal and/or bronchial sampling as described below^102^. Full clinical data and demographics of study participants are detailed in Supplementary Table 2.

### Ethics approval

This study was approved by the Institutional Review Boards (IRBs) of all participating hospitals and institutions and written informed consent was obtained from all participants. Reference numbers pertaining to ethical approvals at each site was as follows: CIRB 2020/2338 (Singapore), IRB-2020-05-004 (Nanyang Technological University, Singapore), X02-0137 (The Sydney South West Area Health Service, Australia) and H-163-1205 (The Hunter New England LHD ethics committee, Australia). For isolation of viruses used in this study, patient samples for cultivation were collected under the guidelines provided by PROTECT (2012/00917) as described previously^103^, a multicentred prospective study to detect novel pathogens and characterize emerging infections, approved by the National Healthcare Group (NHG) as part of pandemic preparedness for national disease outbreaks in Singapore. Work undertaken at the Duke-NUS Medical School Animal Biological Safety Level 3 (ABSL-3) laboratory was approved by the Duke-NUS ABSL3 Biosafety Committee, the National University of Singapore, and the Ministry of Health Singapore (BSL3/2007-03/DA).

### Isolation of human nasopharyngeal epithelial cells (HNPECs)

Nasopharyngeal sampling was performed according to established protocols by trained personnel using Flocked nasopharyngeal swabs (FNPSs) with the BBL Universal Viral Transport Standard Kit. Briefly, a FNPS was inserted into the nostril to an appropriate depth and rotated several times before removal^104^. Samples were immediately transferred on ice to the laboratory for processing. HNPECs were released from swabs by flushing with transport medium at least 20 times. Cells were then plated on human collagen IV pre-coated 6-well plates and cultured in B/D expansion medium (Supplementary Table 3) supplemented with 5 μM of N-[N-(3,5-Difluorophenacetyl)-L-alanyl]-S-phenylglycine t-butyl ester (DAPT) to grow as monolayers. The medium was refreshed every 48 h until confluence was reached.

### Isolation of human bronchial epithelial cells (HBECs)

Bronchial sampling was performed in patients undergoing lung resection, transplantation and/or fibre-optic bronchoscopy according to clinical indications and by standard guidelines as previously described^105,106^. Lung tissue obtained at thoracotomy were dissected and the airways isolated. The epithelium was next removed from the stromal layer by macro-dissection. In samples obtained through bronchoscopy, HBECs were obtained using a single sheathed nylon cytology brush applied under direct vision. Approximately 4-8 brushings were taken from second to third generation bronchi, and cells washed from brushes with DMEM. Cells were then plated in tissue-culture flasks in bronchial epithelial growth medium (BEGM) with growth supplements and medium refreshed every 48 h until confluence was reached.

### Air-liquid interface (ALI) culture

After HNPECs and HBECs reached confluency as monolayers, 2 ×10^5^ cells were seeded into the apical chamber of a 24-well transwell, pre-coated with 30 µg/ml PureCol. The transwell containing the HNPECs or HBECs was first cultured in submerged phase and once the cell layer fully intact, the medium in the apical chamber was removed and cells subsequently cultured at an ALI thereafter. Medium was refreshed twice a week and cells differentiated for 18 days using ALI-Differentiation (ALI-Diff) medium (Supplementary Table 4) and subsequently differentiated into airway organoids.

### NPOs and BOs differentiation

ALI-HNPECs or ALI-HBECs differentiated for 18 days were detached from transwells with TrypLE, resuspended in matrigel and cultured in Airway Organoid (AO) medium (Supplementary Table 5) supplemented with 25% R-spondin-1 conditioned medium, 25 ng/ml FGF7 and 100 ng/ml FGF10 for expansion. Cells were expanded and formed spheroid structures in matrigel. At day 5 expansion, spheroids were passaged into the apical chamber of a 12-well insert for differentiation at ALI for an additional 4-6 weeks. Medium was refreshed twice weekly using AO medium with 5 ng/ml FGF7 and 20 ng/ml FGF10 for 4-6 weeks until organoids were well-differentiated.

### Apical-out NPOs and BOs in suspension culture

Methodologies for the generation of apical-out organoids has been previously reported by others^107,108^. In brief, NPOs and BOs were collected from 12-well inserts and washed with advanced (ad) DMEM/F12 with 100 units/ml penicillin and 100 µg/ml streptomycin (P/S) twice. Washed NPOs and BOs were incubated with 5 mM EDTA in PBS for 1 h at 4 °C with rotation to solubilize the remaining matrigel. NPOs and BOs were centrifuged at 200x *g* for 5 min at 4 °C and supernatant removed. The pellet was re-suspended in medium supplemented with 5 ng/ml FGF7 and 20 ng/ml FGF10 and cultured in suspension using ultra-low attachment 24-well tissue culture plates. Suspended NPOs and BOs were incubated at 37 °C with 5% CO_2_ for 3 days prior to infection studies.

### SARS-CoV-2

Three SARS-CoV-2 strains were used in this study, including (1) an ancestral Wuhan strain from clade L, hCoV-19/Singapore/2/2020 (L-WU) (GISAID accession ID: EPI_ISL_407987); (2) a strain from clade O isolated in mid-2020, hCoV-19/Singapore/1003/2020 (O-614D) (GISAID accession ID: EPI_ISL_574486) and (3) a D614G variant from clade G, hCoV-19/Singapore/1005/2020 (G-614G) (GISAID accession ID: EPI_ISL_574489). Sequence comparison of the three strains is shown in Supplementary Table 1. Virus was propagated in Vero-E6 cells cultured with DMEM supplemented with 5% fetal bovine serum (FBS) and 1% P/S at 37 °C, 5% CO_2_. Cell culture supernatants were harvested, centrifuged and aliquoted once cytopathic effect (CPE) was observed. Viral titres were determined by limited dilution using the Karber method^109^.

### Quantification of live virus by titration

Vero-E6 cells were infected to determine the tissue culture infective dose (TCID_50_/ml). Vero-E6 cells in 96-well plates were utilized for titration assays. Samples were serially diluted in DMEM, supplemented with 5% FBS and 1% penicillin/streptomycin. Cells were infected with 100 µL of diluted sample in quadruplicate. Cells were incubated at 37 °C and 5% CO_2_ for 4 days. Following incubation, wells with CPE were recorded and the cell-free virus titre (TCID_50_/mL) for each sample determined by limited dilution using the Karber method^109^.

### Infection of Vero-E6 cells

Vero-E6 cells were seeded on 24-well tissue culture plates and infected with SARS-CoV-2 at a multiplicity of infection (MOI) of 0.01 to determine viral replication kinetics of the three strains. Supernatants were harvested at 24, 48, 72 and 96 hpi and viral titres determined.

### SARS-CoV-2 infection of NPOs and BOs

Apical-out NPOs and BOs were washed once with adDMEM/F12 with 1% P/S prior to infection and resuspended in 1 ml AO medium. 50 µl of organoid was taken and dissociated with TrypLE express for cell counting and, to determine the total cell number and volume of virus to be used for MOI = 0.1. Apical-out NPOs and BOs were infected with SARS-CoV-2 in suspension on ultra-low attachment plates for 1 h at 37 °C, 5% CO_2_. After 1 h incubation, infected NPOs and BOs were washed with adDMEM/F12 and 1% P/S three times. The organoids were re-embedded in 75% Matrigel and seeded as 30 µl per organoid droplet on a 24-well culture plate. 500 µl of AO medium was added to each respective well after the Matrigel solidified. Cell culture supernatants were then harvested at 1, 24, 48, 72 and 96 hpi for TCID_50_ assay and Luminex assays. Cell lysates were collected in buffer RLT with β-mercaptoethanol at 48 and 72 hpi for quantitative polymerase chain reaction (qPCR) analyses as described below.

### Bacterial culture and infection of NPOs

*Pseudomonas aeruginosa* strain PAO1^110^ was cultured in tryptic soy broth (TSB) with shaking at 37 °C, overnight. *Streptococcus pneumoniae* strain INS-E611 (serotype 6B)^111^ was cultured overnight in Todd Hewitt Broth (THB) at 37 °C and 5% CO_2_. Overnight bacterial cultures were centrifuged and washed with PBS before determination of bacterial concentration by OD_600nm_ measurement. *Pseudomonas aeruginosa* was diluted to a concentration of 5 ×10^6^ CFU/ml and *Streptococcus pneumoniae* diluted to a concentration of 7.5 ×10^6^ CFU/ml in antibiotic-free AO medium prior to infection. After the determination of cell number, apical-out NPOs were infected in suspension for 1 h at 37 °C, 5% CO_2_. After 1 h incubation, infected NPOs were washed with adDMEM/F12 and 1% P/S three times. NPOs were then re-embedded in 75% Matrigel and seeded as 30 µl per organoid droplet on a 24-well culture plate. 500 µl of AO medium (antibiotic-free) with 300 µg/ml gentamicin was added to each respective well after the Matrigel solidified. Cell lysates were collected in buffer RLT with β-mercaptoethanol at 6 hpi for qPCR analyses. Cell culture supernatants were collected at 6 hpi for the detection of secretory cytokines and chemokines using Luminex assays.

### Live and brightfield imaging

Airway organoids (AOs) cultured in matrigel or suspension were imaged and/or videotaped using a Nikon inverted microscope Eclipse Ti-U Nikon inside the ABSL3 facility under the stated magnifications.

### Immunofluorescence microscopy

Intact organoids were fixed in 4% paraformaldehyde for 30 mins at room temperature followed by washing with PBS and permeabilized with 0.2% Triton X-100 in PBS for 30 min. This was followed by blocking with staining buffer (1% BSA, 0.2% Triton X-100 in PBS) for 1 h. Organoids were next incubated with primary antibodies including anti-ACE2, anti-acetylated tubulin, anti-MUC5AC, anti-TP63 and anti-SCGB1A1 diluted in staining buffer for 3 h at room temperature, followed by washing three times with PBS. AlexaFluor 488, 594 or 647-labelled secondary antibodies, phalloidin and 4′,6-diamidino-2-phenylindole (DAPI) were incubated with organoids for 1 h at room temperature. Organoids were mounted on glass slides with ProLong™ Glass antifade mountant and covered with glass coverslips. Images were captured using a Zeiss LSM 800 confocal microscope and processed using ZEN Image analysis software (Zeiss). Information for antibodies used in this study are listed in Supplementary Table 7 and all primary antibodies used in this study were verified ((Supplementary Figures 13, 14).

### qPCR analysis

Total RNA from NPOs and BOs with or without infection respectively were extracted using the RNeasy Plus Mini Kit according to the manufacturer’s instructions. Reverse transcription was then performed using the PrimeScript RT Reagent Kit to generate cDNA under the following conditions: 37 °C for 15 mins, 85 °C for 5 secs followed by 4 °C incubation. cDNA was diluted and amplified using the GoTaq® qPCR SYBR green Master Mix under the following conditions: 95 °C for 2 mins followed by 95 °C for 3 secs and 60 °C for 30 secs for a total of 40 cycles. SARS-CoV-2 N gene was amplified using the GoTaq® Probe qPCR Master Mix under the same conditions. Sequences of primers and probes used in this study are summarised in Supplementary Table 6. qPCR amplification involving SARS-CoV-2 infected samples were performed on a CFX96 Touch real-time PCR detection system (Bio-Rad) inside the ABSL3 facility, while non-SARS-CoV-2 related work was performed on a QuantStudio 6 Flex Real-Time PCR System (Thermofisher Scientific). Absolute quantification was performed for the following genes: *TP63, SCGB1A1, FOXJ1, MUC5AC, ACE2, TMPRSS2, Furin, Neuropilin-1* and the SARS-CoV-2 N gene using standard curves generated with plasmid DNA. Relative amounts of the *P. aeruginosa* 16 S, *S. pneumoniae* lytA, *IL-1β, IL-6, IL-8, IFN- β, TNF-α, CCL2, CCL5* and *CXCL10* mRNA (normalized with β-actin) was determined using the 2^−ΔΔCt^ method^112^.

### Quantification of cytokines and chemokines at protein level

Cell culture supernatants of SARS-CoV-2 infected NPOs and BOs at 48 and 72 hpi and *P. aeruginosa* and *S. pneumoniae*-infected NPOs at 6 hpi were subjected to a multiplexed cytokine assay using a Bio-Plex Pro Human cytokine screening panel (Biorad) containing 48 human cytokines according to manufacturer’s instructions. Spectral intensities were quantified on a MagPix machine (Luminex Corporation) and Bio-Plex 200 System (Bio-Rad) for SARS-CoV-2 and bacterial infection studies, respectively. Cytokine concentrations were calculated by interpolating from standard curves through 5PL curve fitting.

### Micro-optical coherence tomography (µOCT) imaging to visualize and quantify ciliary beat frequency (CBF)

The µOCT technology has been described previously^113–116^. In brief, the µOCT system included a Spectral-domain OCT (SD-OCT) imaging console and a benchtop probe. A supercontinuum light source illuminated a 50/50 beam splitter. Half of the source light was transmitted to the benchtop probe and light returned from the probe was relayed to a spectrometer. The spectrometer was composed of a 960 lines/mm volume phase holographic transmission grating, a multi-element camera lens, and a line scan camera. Light reflected from the reference arm and that scattered back from the sample arm were combined with the beam splitter back to the console. Transverse (x, y) scanning was performed using a pair of galvanometer scanners driven by an analogue output board. The output of the camera was transferred to an image acquisition board.

µOCT imaging was performed on human BOs with illumination incident at the top-side view of the organoids. The imaging optics axis is typically placed within 10° of normal to the cell plane to minimize errors in geometric measurements. Ciliary beat frequency (CBF) is determined using a time series of images and assessed by quantifying the frequency of peak amplitude in the image regions exhibiting oscillatory behaviour. On average 6 to 10 organoids are evaluated per donor in up to 10 regions of ciliary activity per image sequence to determine CBF. All image analysis was performed using ImageJ and MATLAB.

### Single-cell RNA (scRNA) sequencing

#### Cell and library preparation

Single-cell RNA sequencing was performed according to standard protocol of the Chromium Next GEM Single Cell 3′ GEM Library & Gel Bead Kit V3.1 as per manufacturer’s instructions. Briefly, NPOs and BOs were washed once with adDMEM/F12 to eliminate matrigel and then incubated with TrypLE express for 10 min. Single-cell suspensions were next achieved by passing through the large undissociated structures with a P1000 tip repeatedly. Single cells were centrifuged and resuspended in AO medium for cell counting to obtain a density of 1000 cells/µl. Cells were loaded on the Chromium Next GEM chip G and run on the Chromium controller. Sequencing libraries were prepared following the standard manufacturer’s protocol. The DNA libraries were sequenced paired-end and single-indexed on an Illumina HiSeq2500 v2 platform (28x91bp) at the sequencing facility located at the Singapore Centre for Environmental Life Sciences Engineering (SCELSE), Nanyang Technological University, Singapore.

#### Data processing and analysis

Raw fastq reads from NPOs and BOs from non-diseased and COPD were aligned to the human GRCh38 reference genome using Cell Ranger 6.0.2. The single cell count matrices were then analysed with Seurat 4.0^117^. Cells with <200 detected genes and >20% mitochondrial genes were excluded. The filtered datasets were then normalized using the SCTransform function in Seurat and integrated to yield a unified data array for downstream comparative analysis between non-diseased (pooling of one NPO-ND line and one BO-ND line) and COPD (pooling of one NPO-COPD line and one BO-COPD line).

Cell clustering was performed with the resolution parameter set to 0.3. Nonlinear dimensional reduction was used to construct the unified and disease state-specific UMAP plots as illustrated. Differential expression analysis was performed based on non-parametric Wilcoxon rank sum testing with Seurat FindAllMarkers and FindMarkers functions, respectively to identify gene markers of each cell cluster and differentially expressed genes (DEGs) between the non-diseased and COPD cells. Functional enrichment analysis was conducted using Ingenuity Pathway Analysis (Qiagen) and GSEA^118^. To validate our COPD organoid models, we compared the top enriched functions, based on DEGs, from publicly available published bulk RNAseq datasets of cultured airway epithelial cells and/or clinical biopsies from COPD to those from our scRNA-seq datasets^119^. Three relevant datasets (Accession numbers: GSE124180, GSE146532 and GSE162154) were identified and mapped with Rsubread 3.6.2 to Ensembl gene ID^120^, however, GSE124180 and GSE146532 had to be excluded because their non-diseased and COPD sample sets did not form distinct clusters from each other based on principal component analysis (PCA). Consequently, only GSE162154 was used for our comparative analysis. The bulk RNAseq datasets were downloaded from Gene Expression Omnibus (GEO).

Cell type annotation was first analysed using Blueprint, ENCODE and the Human Primary Cell Atlas databases using celldex (1.2.0) which confirmed that all cells exhibited an epithelial phenotype^121^. To determine the cell type of each respective cluster, GSEA was used to make comparisons to the airway and lung cell signature gene sets respectively from Garcia et al. (2019)^36^ and Travaglini et al. (2020)^37^ respectively. Pseudotime analysis was performed using Monocle3^122^ and cell trajectory inferred with the cycling basal cells specified as the root node (origin of the trajectory; pseudotime = 0).

### Bulk RNA sequencing

Non-diseased and COPD NPOs were either mock-treated or infected with *Pseudomonas aeruginosa* (PAO1) for 6 h. Organoids were then lysed with Qiagen RLT plus buffer and RNA isolated using Qiagen RNeasy Plus Mini Kit. Stranded mRNA libraries for each sample was prepared with oligo dT purification using the Illumina TruSeq Stranded mRNA Kit. Libraries were sequenced paired-end 100 bp (PE100) using Illumina HiSeq2500 System.

Raw paired-end sequencing data in FASTQ format (available in NCBI’s Gene Expression Omnibus (GEO) database: GSE201465 were trimmed for adaptor sequence/low-quality sequence using trimmomatic-0.39 (parameter: /trimmomatic-0.39/adapters/TruSeq3-PE-2.fa:2:30:10:1:TRUE LEADING:3 TRAILING:3 SLIDINGWINDOW:4:15 MINLEN:30). Quality of sequence reads after trimming was checked using FastQC v0.11.8. Trimmed sequence reads were then mapped to the human reference genome GRCh38.p13 (hg38) using the align function available in Rsubread v2.6.4, which was run in R v4.1.0 software (parameter: Type=rna). Feature count extraction was performed using featureCounts function available in Rsubread v2.6.4, which was then run in the R v4.1.0 software (parameters: annot.ext=hg38.ensGene.gtf, isGTFAnnotationFile=TRUE, isPairedEnd=TRUE).

Differential gene expression was performed using DESeq2 (v1.34.0)^123^. Genes were considered differentially expressed when the log2 Fold Change >±  1 and adjusted p-value was <0.05. All differentially expressed genes (DEGs) between mock-treated and *P.aeruginosa*-infected non-diseased and COPD NPOs were subject to hierarchical clustering based on their expression trend to form 5 major gene clusters. The enriched gene ontology terms of each gene cluster was analyzed using ViSEAGO^124^.

### Statistical analysis

For non-infection related experiments described in Figs. 1–3, *n* = 7; *n* = 10; *n* = 3 and *n* = 8 biologically independent replicates were used for NPO-non diseased (ND); NPO-COPD; BO-ND and BO-COPD, respectively. For infection studies described in Figs. 4–6, at least *n* = 3 biologically independent replicates were performed. Data were analyzed using GraphPad Prism software (version 8.3.0). Continuous data were tested for normality using the Kolmogorov-Smirnoff test (K-S test). Data are presented as mean with standard error for normally distributed data, and a Student *t* test or analysis of variance (ANOVA) used to assess for inter-group differences as appropriate. For non-normal variables, medians are presented with interquartile range, and the Wilcoxon signed rank test (paired data) and Mann-Whitney U test (unpaired data) performed when comparing two groups and/or the Kruskal-Wallis test for > 2 groups, respectively. Spearman’s correlation coefficient (nonparametric) analyses were performed to assess for correlations between specific gene expression levels and forced expiratory volume in the 1st second percent predicted (FEV_1_% predicted). A *p-*value <0.05 was considered significant and degree of significance illustrated as follows: *P < 0.05; **P < 0.01; ***P < 0.001 and ****P < 0.0001.

### Reporting summary

Further information on research design is available in the Nature Portfolio Reporting Summary linked to this article.

## Supplementary information

Supplementary Information

Description of Additional Supplementary Information

Supplementary Data 1

Supplementary Video 1

Supplementary Video 2

Supplementary Video 3

Reporting Summary

## Data Availability

Single cell RNA sequencing: Raw fastq reads from NPOs and BOs from non-diseased and COPD were aligned to the human GRCh38. p13 (hg38) reference genome. Three publicly available published bulk RNAseq datasets ((Accession numbers: GSE124180, GSE146532 and GSE162154) of cultured airway epithelial cells and/or clinical biopsies from COPD were used for comparison with our scRNA dataset. The 10× single-cell RNA sequencing data generated in this study has been deposited in the GEO database under the accession code GSE186017. Bulk RNA sequencing: Raw FASTQ reads were mapped to the human reference genome GRCh38.p13 (hg38). The bulk RNA sequencing data generated in this study has deposited in the GEO database under the accession code GSE201465. All other relevant data supporting the key findings of this study are available within the article and its supplementary information files. Source data is provided in this paper. Source data are provided with this paper.

## Source data

Source Data

## Code availability

R scripts for single-cell RNA-seq analysis is available at https://github.com/chenghongsheng/SC_RNAseq-airway-organoid and 10.5281/zenodo.7290276.
