# Supplementary material for: The establishment of COPD organoids to study host-pathogen interaction reveals enhanced viral fitness of SARS-CoV-2 in bronchi
Source: Nat Commun. 2022 Dec 10;13:7635. doi: 10.1038/s41467-022-35253-x (PMC9735280; doi:10.1038/s41467-022-35253-x)
Supplement: Supplementary file 1 — Supplementary Information [file 41467_2022_35253_MOESM1_ESM.docx]

**Supplementary information**

**The establishment of COPD organoids to study host-pathogen interaction reveals enhanced viral fitness of SARS-CoV-2 in bronchi**

Louisa L.Y. Chan, Danielle E. Anderson, Hong Sheng Cheng, Fransiskus Xaverius Ivan, Si Chen, Adrian E.Z. Kang, Randy Foo, Akshamal M. Gamage, Pei Yee Tiew, Mariko Siyue Koh, Ken Cheah Hooi Lee, Kristy Nichol, Prabuddha S. Pathinayake, Yik Lung Chan, Tsin Wen Yeo, Brian G. Oliver, Peter A.B. Wark, Linbo Liu, Nguan Soon Tan, Lin-Fa Wang, Sanjay H. Chotirmall

**Supplementary Figure 1. Characterization of nasopharyngeal and bronchial organoids from non-diseased individuals and chronic obstructive pulmonary disease (COPD).** **A-B.** Immunofluorescence staining of human (A) nasopharyngeal organoids (NPOs) (upper airway) and (B) bronchial organoids (BOs) (lower airway) from non-diseased individuals (left) and patients with COPD (right) for TP63 (basal cells: green), SCGB1A1 (club cells: purple) and acetylated-α-tubulin (Ac-tubulin) (ciliated cells: green). Nuclei and F-actin are counterstained with DAPI (blue) and Phalloidin (red/white) respectively. Scale bar = 20 μm. Data are representative of at least 5 independent experiments.

**Supplementary Figure 2. Exacerbation frequency and lung function do not associate with TP63, SCGB1A1 and FOXJ1 gene levels in nasopharyngeal and bronchial organoids in COPD.** qRT-PCR analysis of total RNA extracted from NPOs and BOs derived from non-diseased individuals (ND) and COPD for *TP63* (basal cells), *SCGB1A1* (club cells) and *FOXJ1* (ciliated cells) are illustrated left to right. **A-C**. qRT-PCR analysis of total RNA extracted from NPOs and BOs derived from ND and COPD for (A) *TP63*, (B) *SCGB1A1* and (C) *FOXJ1*. ND and COPD of GOLD stage B, C and D are denoted by black, blue, yellow, and green symbols, respectively. n=7; n=10; n=3 and n=8 biologically independent experiments for NPO-ND; NPO-COPD; BO-ND and BO-COPD are illustrated. Data are presented as medians ± interquartile range and the Mann-Whitney U test was performed. **P* < 0.05; ***P* < 0.01. [*TP63*: NPO-ND vs NPO-COPD= 0.0431; NPO-ND vs BO-ND= 0.0333; *SCGB1A1:* NPO-ND vs BO-ND= 0.0238; BO-ND vs BO-COPD= 0.0238; *FOXJ1:* NPO-ND vs BO-ND= 0.0238; BO-ND vs BO-COPD= 0.0242]. Gene expression is stratified according to **D-F**. exacerbation frequency as non-exacerbator (NE); exacerbator (E) or frequent-exacerbator (FE) and **H-J**. Lung function as forced expiratory volume in the 1^st^ second percent predicted (FEV1 % predicted). n=28 biologically independent samples are illustrated. Data are presented as medians ± interquartile range and the Mann-Whitney U test is performed. **P* < 0.05. [Exacerbations: *TP63*: ND vs FE= 0.0330] [FEV1 % predicted: *TP63*: ND vs 50%-79%= 0.0235] **K-M**. Scatter plots of (K) *TP63*, (L) *SCGB1A1* and (M) *FOXJ1* gene expression in COPD organoids according to FEV1 % predicted. n=28 biologically independent samples are illustrated. Spearman’s correlation coefficient (non-parametric) analysis was performed. [*TP63: P=*0.7110*; SCGB1A1: P=*0.4430*; FOXJ1: P=*0.2535] Source data are provided as a Source Data file.

**Supplementary Figure 3. Single cell RNA-seq reveals developmental and functional impairment in COPD organoids. A.** Unified UMAP plots of scRNA-seq transcriptomic data highlight the main cell types detected in lung organoids of non-diseased (ND) and COPD (n=19,877 cells). **B.** Dot plot illustrating the expression profiles of cell type-specific markers for the main cell types detected in lung organoids. Dot size indicates proportion of cells expressing a particular gene while colour intensity indicates level of expression from low to high as grey to red. **C.** Individual UMAP plots (top panel) and stacked bar plot of the relative abundance of cell types (bottom panel) in the NPO-ND (3974 cells), BO-ND (4592 cells), NPO-COPD (3611 cells) and BO-COPD (7652 cells). **D.** Ridgeline plots showing the expression distribution of *TP63*, *SCGB1A1*, and *FOXJ1* in different cell types from the ND and COPD airway organoids. Vertical lines indicate the median expression. The cell types in which *TP63*, *SCGB1A1*, and *FOXJ1* serve as the key biomarkers respectively are highlighted in red.

**
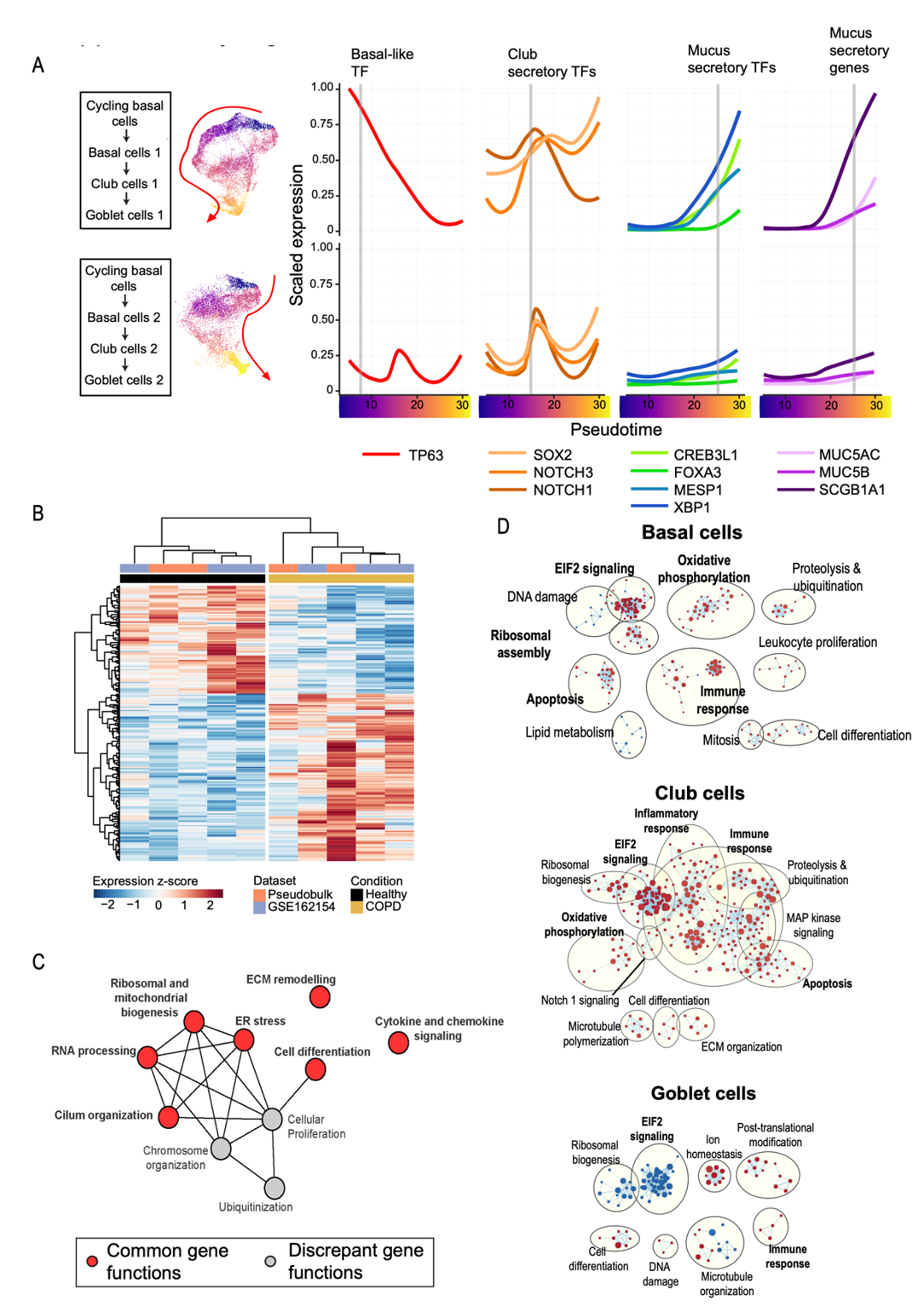
**

**Supplementary Figure 4. Functional analysis of ND and COPD airway organoids. A.** Scaled, smoothed expression profiles of key transcription factors (TFs) and markers across the pseudotime of goblet cells differentiation along the normal (top panel) and impaired (bottom panel; enriched in COPD airway organoids) trajectories. **B.** Heatmap illustrating the expression profiles of differentially expressed genes between healthy and COPD organoids of our scRNAseq data and the publicly available bulk-RNAseq datasets derived from cultured human small airway epithelia in COPD (GEO Accession number: GSE162154). The scRNAseq data were converted to pseudobulk to identify the differentially expressed genes. **C.** Common and discrepant enriched gene functions of COPD (relative to ND) airway organoids between the pseudobulk data of our scRNAseq data and the bulk-RNAseq datasets derived from GSE162154. **D.** The linkage networks derived from GSEA are illustrated for the gene sets from basal (top panel), club (middle panel) and goblet cells (bottom panel) in COPD compared to ND organoids. Each node represents one gene set and colouration denotes gene activation status where red nodes indicate gene activation and blue nodes gene inactivation. Nodes with similar function cluster together and their biological functions annotated.

**Supplementary Figure 5.** **Lung organoids express SARS-CoV-2 entry factors. A-D.** qRT-PCR analysis of total RNA extracted from nasopharyngeal (NPOs) and bronchial (BOs) organoids respectively derived from non-diseased individuals (ND) and COPD for (A) Angiotensin converting enzyme-2 (*ACE2*), (B) Transmembrane protease serine 2 (*TMPRSS2*) (C) *Furin* and (D) *Neuropilin-1*. COPD patients of GOLD stages B, C and D are denoted by brown, green and purple symbols respectively. n=7; n=10; n=3 and n=8 biologically independent experiments for NPO-ND; NPO-COPD; BO-ND and BO-COPD are illustrated. Data are presented as medians ± interquartile range and the Mann-Whitney U test was performed. **P* < 0.05; ****P* < 0.001. [*ACE2*: NPO-ND vs NPO-COPD=0.0330; BO-ND vs BO-COPD=0.0121; NPO-ND vs BO-ND=0.0167; *TMPRSS2*: NPO-ND vs NPO-COPD= 0.0002; BO-ND vs BO-COPD= 0.0242; NPO-ND vs BO-ND=0.0167; *Neuropilin-1* :NPO-ND vs BO-ND= 0.0333] **E-L**. (E and I) *ACE2*, (F and J) *TMPRSS2*, (G and K) *Furin* and (H and L) *Neuropilin-1* gene expression in NPOs and BOs derived from COPD and stratified according to **(E-H)** exacerbation frequency as non-exacerbator (NE); exacerbator (E) and frequent-exacerbator (FE) and **(I-L)** lung function as forced expiratory volume in the 1^st^ second percent predicted (FEV_1_ % predicted). n=28 biologically independent samples are illustrated. Data are presented as medians ± interquartile range and the Mann-Whitney U test was performed. **P* < 0.05; ***P* < 0.01; ****P* < 0.001. [Exacerbations: *ACE2*: ND vs E= 0.0173; ND vs FE= 0.0064; *TMPRSS2:* ND vs E= 0.0049; ND vs FE= 0.0159; *Furin:* ND vs NE= 0.0440; ND vs E= 0.0045; ND vs FE= 0.0022; *Neuropilin-1:* ND vs E= 0.0025] [FEV_1_ % predicted: *TMPRSS2:* ND vs <30%= 0.0248; *Furin:* ND vs 30%-49%= 0.0005; ND vs <30%= 0.0011; *Neuropilin-1:* ND vs <30%= 0.0007]**M-P.** Scatter plots of (M) ACE2, (N) TMPRSS2, (O) Furin and (P) Neuropilin-1 gene expression in COPD organoids according to FEV_1_ % predicted. n=28 biologically independent samples are illustrated. Spearman’s correlation coefficient (non-parametric) analysis was performed. *P*=0.1067 (*ACE2*); *P*=0.3406 (*TMPRSS2*); *P*=0.0072 (*Furin*); *P*=0.0503 (*Neuropilin-1*). Source data are provided as a Source Data file.

**Supplementary Figure 6. COPD airway organoids express SARS-CoV-2 entry factor-related genes. A.** Spatial expression of *ACE2*, *TMPRSS2, FURIN* and *NRP1* in the airway organoids. **B.** Distribution (left panel) and relative abundance (right panel) of *ACE2^+^, TMPRSS2^+^, NRP1^+^/ FURIN*^+^, and *TMPRSS2^+^/ NRP1^+^/ FURIN^+^* cells across the cellular subpopulations in ND and COPD airway organoids.

**
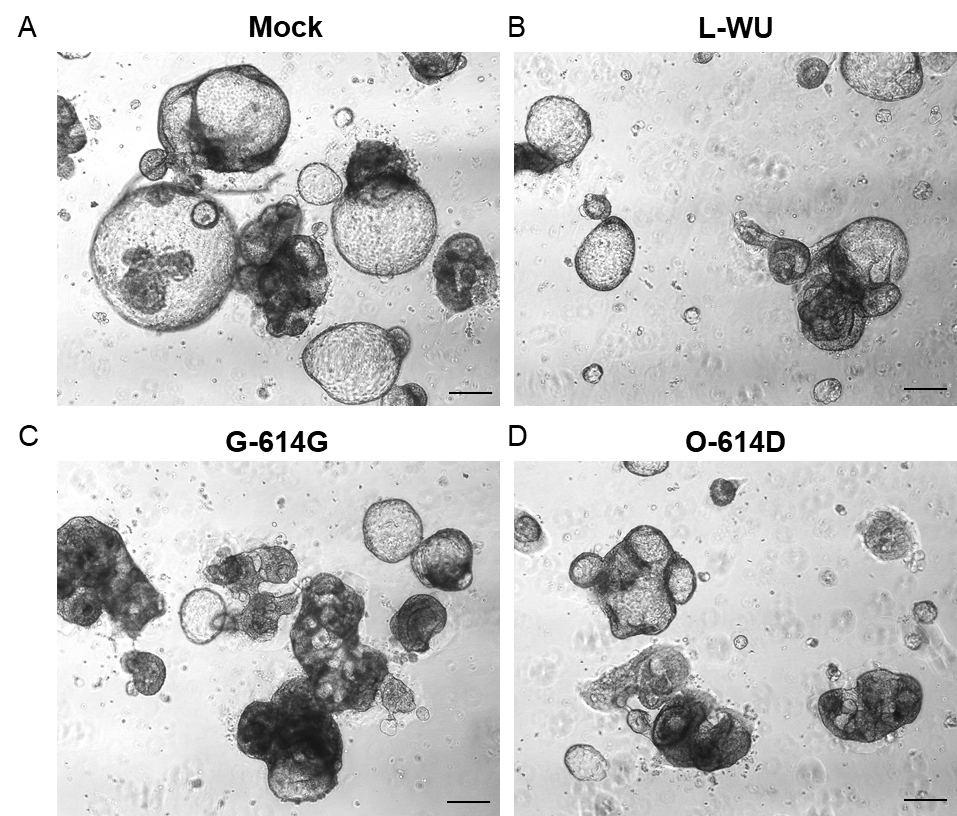
**

**Supplementary Figure 7. Cytopathological effect of SARS-CoV-2 infected human nasopharyngeal organoids.** **A-D.** Representative brightfield images of human nasopharyngeal organoids (NPOs) following (A) mock infection, (B) SARS-CoV-2 (L-WU) infection (C) SARS-CoV-2 (G-614G) infection and (D) SARS-CoV-2 (O-614D) infection. Scale bar = 100 μm. Data are representative of 3 independent experiments.

**Supplementary Figure 8. Nasopharyngeal organoids (NPOs) exhibit higher SARS-CoV-2 replication competence for the ancestral Wuhan (L-WU) and D614G strains. A-C.** qRT-PCR analysis of total RNA extracted from SARS-CoV-2 (A) L-WU (B) G-614G and (C) O-614D infected NPOs (solid bars-left sided) and BOs (lined bars-right sided) respectively at 48 and 72 hpi for nucleocapsid gene expression. n=4 and n=5 biologically independent experiments for NPO and BO infection respectively are illustrated. Data are presented as mean ± SEM. **D-F.** Viral replication kinetics from culture supernatants harvested from SARS-CoV-2 (D) L-WU, (E) G-614G and (F) O-614D-infected NPOs and BOs respectively at 1, 24, 48, 72 and 96 hpi by TCID_50_ assay. n=4 biologically independent experiments are illustrated. Data are presented as mean ± SEM and unpaired t test was performed. **P* < 0.05. [L-WU= 0.0202; G-614G=0.0475] **G.** Viral replication kinetics from culture supernatants harvested from SARS-CoV-2 L-WU, G-614G and O-614D-infected NPOs at 168 hpi by TCID_50_ assay. n=3 biologically independent experiments are illustrated. Data are presented as mean ± SEM and one-way ANOVA was performed. **P* < 0.05. [L-WU vs G-614G= 0.0210; G-614G vs O-614D= 0.0210] **H.** CXCL10 released upon SARS-CoV-2 infection in NPOs and BOs at 48 and 72 hpi by multiplex Luminex assay. n=3 biologically independent experiments are illustrated. Data are presented as mean ± SEM and one-way ANOVA was performed. **P* < 0.05. [NPO: 48 hpi: MK vs L-WU=0.0175; L-WU vs O-614D=0.0470; 72 hpi: MK vs G-614G= 0.0132; G-614G vs O-614D= 0.0290] [BO: 72 hpi: MK vs G-614G= 0.0459] Source data are provided as a Source Data file.

**Supplementary Figure 9. Cytokines and chemokines secreted from SARS-CoV-2 infected nasopharyngeal organoids (NPOs) and bronchial organoids (BOs).** CCL2, TRAIL, IL-1RA, IL-6, IL-8, TNF-β, MIF, MIP-1β and GRO-α released upon SARS-CoV-2 infection in NPOs (A) and BOs (B) at 48 and 72 hpi by multiplex Luminex assay. n=3 biologically independent experiments are presented. Data are presented as mean ± SEM. Source data are provided as a Source Data file.

**Supplementary Figure 10. Viral replication kinetics of SARS-CoV-2 in Vero-E6 cells.** Viral replication kinetics from culture supernatants harvested from SARS-CoV-2 L-WU (red line), G-614G (green line) and O-614D (purple line) infected Vero-E6 cells (MOI=0.01) at 24, 48, 72 and 96 hpi by TCID_50_ assay. n=3 biologically independent experiments for L-WU and G-614G and n=2 biologically independent experiments for O-614D. Data are presented as mean ± SEM. Source data are provided as a Source Data file.

**Supplementary Figure 11. SARS-CoV-2 infectivity in non-diseased and COPD organoids. A-D.** qRT-PCR analysis of total RNA extracted from SARS-CoV-2 (A-B) L-WU-infected and (C-D) G-614G -infected NPOs (A and C) and BOs (B and D) derived from non-diseased individuals and COPD patients respectively at 48 and 72 hpi for nucleocapsid gene expression. n=5 and n=3 biologically independent experiments for non-diseased and COPD infection are illustrated, respectively. Data are presented as means ± SEM. Source data are provided as a Source Data file.

**Supplementary Figure 12. Nasopharyngeal organoids are permissive to bacterial infection by *Streptococcus pneumoniae.* A.** qRT-PCR analysis of total RNA extracted from *S. pneumoniae*-infected NPOs derived from non-diseased individuals and COPD patients at 6 hpi for the *S. pneumoniae* lytA gene. n=4 for ND and n=6 for COPD. Data are presented as means ± SEM. **B.** qRT-PCR analysis of *S. pneumoniae*-infected NPOs at 6 hpi for *CCL2, CCL5, CXCL10, TNF-α, IL-1β, IL-6 and IL-8*. n=4 and n=6 biologically independent experiments for non-diseased and COPD are illustrated. Data are presented as means ± SEM and unpaired t-test is performed. **P* < 0.05. [*IL-8*= 0.0207]**C.** TNF-β, IFN-γ, macrophage migration inhibitory factor (MIF), IL-6 and IL-8 release upon *S. pneumoniae* infection in NPOs at 6 hpi by multiplex Luminex assay. n=4 and n=6 biologically independent experiments for non-diseased and COPD are illustrated. Data are presented as means ± SEM and unpaired t-test is performed. **P* < 0.05; ***P* < 0.01; ****P* < 0.001. [TNF-β: ND-MK vs ND-SP= 0.0444; IFN-γ: COPD-MK vs COPD-SP= 0.0019; MIF: ND-MK vs ND-SP= 0.0034; COPD-MK vs COPD-SP= 0.0009; ND-SP vs COPD-SP= 0.0395; IL-8: ND-MK vs ND-SP= 0.0053; COPD-MK vs COPD-SP= 0.0385; ND-SP vs COPD-SP= 0.0171] Source data are provided as a Source Data file.


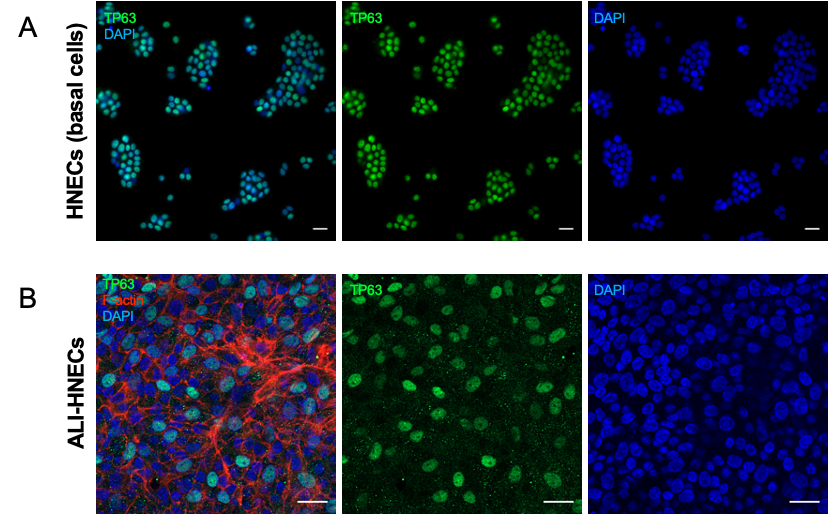


**Supplementary Figure 13. Verification of anti-TP63 antibody by immunostaining of HNECs and ALI-HNECs.** Immunofluorescence staining of HNECs (A) and ALI-HNECs (B) for TP63. Nuclei and F-actin are counterstained with DAPI. Scale bar = 20 μm. Data are representative of 3 independent experiments.


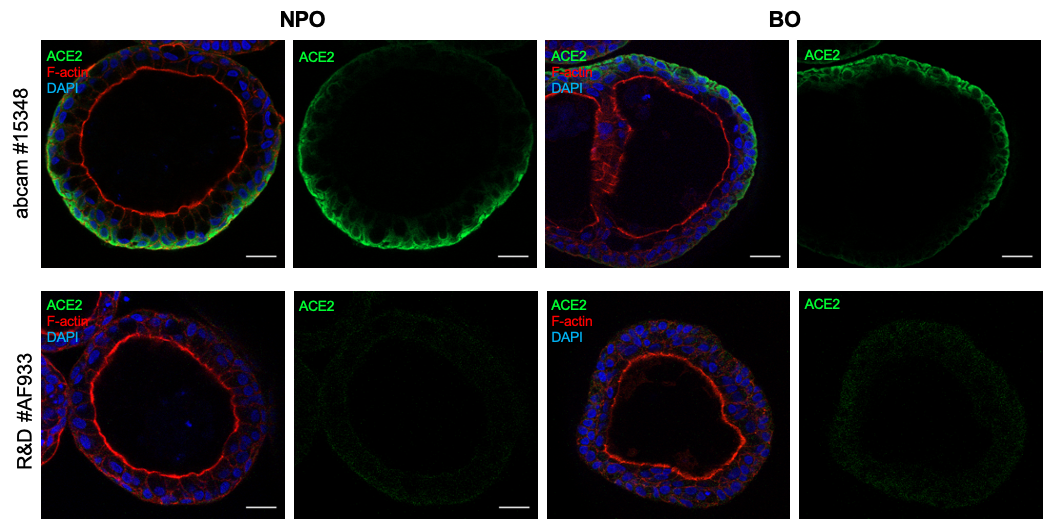


**Supplementary Figure 14. Verification of anti-ACE2 antibodies by immunostaining of NPOs and BOs.** Immunofluorescence staining of NPOs and BOs for ACE2 with abcam #15348 and R&D #AF933 antibodies. Nuclei and F-actin are counterstained with DAPI. Scale bar = 20 μm. Data are representative of 3 independent experiments.

**Supplementary Table 1. Nucleotide sequence and amino acid differences between the SARS-CoV-2 strains used in this study.**

| **Position** | **Gene** | **Codon change** | | | **Protein change** | **Protein change type** |
| --- | --- | --- | --- | --- | --- | --- |
|  |  | **hCoV-19/SG/2/2020**  **(L-WU)** | **hCoV-19/SG/1003/2020**  **(O-614D)** | **hCoV-19/SG/1005/2020**  **(G-614G)** |  |  |
| 1627 | ORF1a (NSP2) | CTT | CTY (mixed) | CTT | L274L | synonymous |
| 3037 | ORF1a (NSP3) | TTC | TTC | TTT | F106F | synonymous |
| 6310 | ORF1a (NSP3) | AGC | AGA | AGC | S1197R | Non-synonymous |
| 6312 | ORF1a (NSP3) | ACA | AAA | ACA | T1198K | Non-synonymous |
| 11083 | ORF1a (NSP6) | TTG | TTT | TTG | L37F | Non-synonymous |
| 13730 | ORF1b (NSP12) | GCT | GTT | GCT | A97V | Non-synonymous |
| 14408 | ORF1b (NSP12) | CCT | CCT | CTT | P323L | Non-synonymous |
| 19524 | ORF1b (NSP15) | CTC | CTT | CTC | L495L | synonymous |
| 23185 | Spike glycoprotein | TTC | TTT | TTC | F541F | synonymous |
| 23403 | Spike glycoprotein | GAT | GAT | GGT | D614G | Non-synonymous |
| 23929 | Spike glycoprotein | TAC | TAT | TAC | Y789Y | synonymous |
| 27147 | ORF5 (M) | CAC | GAC | GAC | D209H | Non-synonymous |
| 27238 | ORF6 (NS6) | GAG | GAG | TAG | E13stop | Non-synonymous |
| 27493 | ORF7a (NS7a) | CCT | YCT (mixed) | CCT | P34X | Non-synonymous |
| 28311 | ORF9 (N) | CCC | CTC | CCC | P13L | Non-synonymous |

**Supplementary Table 2. Clinical data and participant demographics of the study population (n=28).**

| **Sample type** | **Age** | **Sex** | **Data presented in figures** | **Smoking status** | **Non-diseased (ND) or COPD** | **Lung function (as FEV1% predicted)** | **GOLD stage** | **Exacerbation frequency** | **COPD Medication** | | | | | |
| --- | --- | --- | --- | --- | --- | --- | --- | --- | --- | --- | --- | --- | --- | --- |
|  |  |  |  |  |  |  |  |  | **LAMA** | **LABA** | **ICS** | **LABA/LAMA** | **LABA/ICS** | **Triple therapy** |
| Nasopharyngeal | 34 | M | 1, 2A-F, 3 (scRNA), 4, 5, 6 (bulkRNAseq) | Never | ND | N/A | N/A | N/A | N/A | | | | | |
| Nasopharyngeal | 42 | M | 1, 2A-F, 4, 5 | Ex-smoker | ND | N/A | N/A | N/A | N/A | | | | | |
| Nasopharyngeal | 23 | M | 1, 2A-F | Never | ND | N/A | N/A | N/A | N/A | | | | | |
| Nasopharyngeal | 27 | F | 1, 2A-F, 4, 5, 6 | Never | ND | N/A | N/A | N/A | N/A | | | | | |
| Nasopharyngeal | 25 | F | 1, 2A-F, 4, 5, 6 (bulkRNAseq) | Never | ND | N/A | N/A | N/A | N/A | | | | | |
| Nasopharyngeal | 25 | F | 1, 2A-F, 4, 5, 6 (bulkRNAseq) | Never | ND | N/A | N/A | N/A | N/A | | | | | |
| Nasopharyngeal | 52 | M | 1, 2A-F, 4 | Never | ND | N/A | N/A | N/A | N/A | | | | | |
| Nasopharyngeal | 64 | M | 2A-F, 5, 6 (bulkRNAseq) | Ex-smoker | COPD | 46 | D | ⩾2 (FE) | N | N | N | N | N | Y |
| Nasopharyngeal | 64 | M | 2A-F, 3 (scRNA), 5, 6 (bulkRNAseq) | Ex-smoker | COPD | 19 | D | ⩾2 (FE) | N | N | N | N | N | Y |
| Nasopharyngeal | 71 | M | 2A-F, 5, 6 (bulkRNAseq) | Ex-smoker | COPD | 67 | D | ⩾2 (FE) | N | N | N | N | N | Y |
| Nasopharyngeal | 78 | M | 2A-F, 6 | Ex-smoker | COPD | 53 | C | 1 (E) | N | N | N | Y | N | N |
| Nasopharyngeal | 49 | M | 2A-F | Ex-smoker | COPD | 65 | C | 1 (E) | N | N | N | N | N | Y |
| Nasopharyngeal | 79 | M | 2A-F | Current smoker | COPD | 62 | B | 0 (NE) | N | N | N | Y | N | N |
| Nasopharyngeal | 61 | M | 2A-F, 6 | Current smoker | COPD | 42 | D | ⩾2 (FE) | N | N | N | N | N | Y |
| Nasopharyngeal | 75 | M | 2A-F, 6 | Ex-smoker | COPD | 37 | D | 1 (E) | N | N | N | N | N | Y |
| Nasopharyngeal | 86 | M | 2A-F | Ex-smoker | COPD | 58 | C | ⩾2 (FE) | N | N | N | N | N | Y |
| Nasopharyngeal | 63 | M | 2A-F, 6 | Ex-smoker | COPD | 38 | B | 0 (NE) | N | N | N | N | N | Y |
| Bronchial | 59 | M | 1, 2A-F, 2G-H, 3 (scRNA), 4, 5 | Never | ND | N/A | N/A | N/A | N/A | | | | | |
| Bronchial | 55 | M | 1, 2A-F, 2G-H, 4, 5 | Never | ND | N/A | N/A | N/A | N/A | | | | | |
| Bronchial | 54 | F | 1, 2A-F, 2G-H, 4, 5 | Never | ND | N/A | N/A | N/A | N/A | | | | | |
| Bronchial | 74 | F | 2A-F, 3 (scRNA), 5 | Never | COPD | 53 | C | 1 (E) | Y | N | N | N | N | N |
| Bronchial | 79 | M | 2A-F, 2G-H, 5 | Never | COPD | 63 | C | 1 (E) | N | N | N | N | N | Y |
| Bronchial | 84 | M | 2A-F, 2G-H, 5 | Never | COPD | 61 | C | 1 (E) | N | Y | N | N | N | N |
| Bronchial | 78 | M | 2A-F | Current smoker | COPD | 61 | C | 1 (E) | N | N | N | N | N | Y |
| Bronchial | 70 | M | 2A-F | Current smoker | COPD | 62 | C | 1 (E) | N | N | Y | N | N | N |
| Bronchial | 81 | F | 2A-F, 2G-H | Current smoker | COPD | 74 | C | 1 (E) | Y | N | N | N | N | N |
| Bronchial | 59 | F | 2A-F | Ex-smoker | COPD | 25 | D | ⩾2 (FE) | Y | N | N | N | Y | N |
| Bronchial | 64 | F | 2A-F | Current smoker | COPD | 25 | D | ⩾2 (FE) | Y | N | N | N | Y | N |

N/A: Not applicable; N: No; Y: Yes; M: Male; F: Female; NE: Non-exacerbator; E: Exacerbator; FE: Frequent-exacerbator

**Supplementary Table 3. B/D expansion medium.**

| **Name of reagent or supplement** | **Brand** | **Cat#** | **Final Concentration** |
| --- | --- | --- | --- |
| Bronchial epithelial cell medium | Sciencell | 3211 | 50% |
| Advanced (ad) DMEM/F12 | Gibco | 12634010 | 23.5% |
| R-spondin 1 conditioned medium | In-house preparation | | 20% |
| HEPES | Gibco | 15630080 | 10 mM |
| Glutamax | Invitrogen | 35050-61 | 2.0 mM |
| P/S | Gibco | 15070-063 | 100 U/ml |
| Primicon | Invivogen | Ant-pm-2 | 100 U/ml |
| B27 | Gibco | 12587010 | 2% |
| Hydrocortisone | Sigma | H0888 | 0.5 μg/ml |
| 3,3’,5-Triido-L-thyronine (T3) | Sigma | T6397 | 100 nm |
| Epinephrine | Sigma | E4642 | 0.5 μg/ml |
| N-acetyl-cysteine | Sigma | A9165 | 1.25 mM |
| NicotiN/Amide | Sigma | N0636 | 5 mM |
| TGFb inhibitor (A83-01) | Tocris | 2939 | 1 μM |
| BMPi (DMH-1) | Selleckem | S7146 | 1 μM |
| Rocki (Y-27632) | Abmole bioscience | Y-27632 | 5 μM |
| SB202190 | Sigma | S7067 | 500 nM |
| Fibroblast growth factor 10 (FGF10) | Peprotech | 100-26 | 100 ng/ml |
| Fibroblast growth factor 7 (FGF7) | Peprotech | 100-19 | 25 ng/ml |
| Insulin-like growth factor-1 (IGF-1) | Peprotech | 100-11 | 25 ng/ml |

**Supplementary Table 4. ALI-Differentiation (ALI-Diff) medium.**

| **Name of reagent or supplement** | **Brand** | **Cat#** | **Final Concentration** |
| --- | --- | --- | --- |
| Advanced (ad) DMEM/F12 | Gibco | 12634-010 | 1x |
| Hydrocortisone | Sigma | H088 | 0.5 μg/ml |
| 3,3’,5-Triido-L-thyronine (T3) | Sigma | T6397 | 100 nM |
| Epinephrine | Sigma | E4642-5g | 0.5 μg/ml |
| hEGF | Thermofisher | PHG0311 | 0.5 ng/ml |
| TTNPB | Cayman | No.16144 | 100 nM |
| A83-01 | Tocris | 2939/10 | 50 nM |
| P/S | Gibco | 15070063 | 100 U/ml |

**Supplementary Table 5. Airway Organoid (AO) medium.**

| **Name of reagent or supplement** | **Brand** | **Cat#** | **Final Concentration** |
| --- | --- | --- | --- |
| R-spondin 1 conditioned medium | In-house preparation | | 25% or 0% |
| FGF7 | Peprotech | 100-19 | 25 ng/ml or 5 ng/ml |
| FGF10 | Preptotech | 100-26 | 100 ng/ml or 25 ng/ml |
| A83-01 | Tocris | 2939 | 500 nM |
| Y-27632 | Abmole | Y-27632 | 5 μM |
| SB202190 | Sigma | S7067 | 500 nM |
| B27 | Gibco | 17504-44 | 1x |
| N-acetyl-cysteine | Sigma | A9165-5g | 1.25 mM |
| NicotiN/Amide | Sigma | N0636 | 5 mM |
| GlutaMax | Invitrogen | 12634-034 | 1x |
| HEPES | Invitrogen | 15630-056 | 10 mM |
| P/S | Gibco | 15070-063 | 100 U/ml |
| Primocin | Invivogen | Ant-pm-1 | 50 μg/ml |
| Advanced (ad) DMEM/F12 | Invitrogen | 12634-034 | 1x |

**Supplementary Table 6. qPCR primers and probes used in this study.**

|  | **5'--> 3' sequence** |
| --- | --- |
| Human *MUC5AC* F | GGAACTGTGGGGACAGCTCTT |
| Human *MUC5AC* R | GTCACATTCCTCAGCGAGGTC |
| Human *Beta-actin* F | TGGATCAGCAAGCAGGAGTATG |
| Human *Beta-actin* R | GCATTTGCGGTGGACGAT |
| Human *FOXJ1* F | CTTTCAAGAAGCGGCGACTG |
| Human *FOXJ1* R | CCTCGGTATTCACCGTCAGC |
| Human *TP63* F | CCCGTTTCGTCAGAACACAC |
| Human *TP63* R | CATAAGTCTCACGGCCCCTC |
| Human *SCGB1A1* F | ATGAAACTCGCTGTCACCCT |
| Human *SCGB1A1* R | GGGTTTCGATGACACGCTGA |
| Human *IFN-β* F | CAACTTGCTTGGATTCCTACAAAG |
| Human *IFN-β* R | TGCCACAGGAGCTTCTGACA |
| Human *IL-1β* F | CACGATGCACCTGTACGATCA |
| Human *IL-1β* R | GTTGCTCCATATCCTGTCCCT |
| Human *IL-6* F | GCATGGGCACCTCAGATTGT |
| Human *IL-6* R | TGCCCAGTGGACAGGTTTCT |
| Human *IL-8* F | CACCGGAAGGAACCATCTCA |
| Human *IL-8* R | AGAGCCACGGCCAGCTT |
| Human *TNF-α* F | GCAGGTCTACTTTGGGATCATTG |
| Human *TNF-α* R | GCGTTTGGGAAGGTTGGA |
| Human *CCL2* F | CAAGCAGAAGTGGGTTCAGGAT |
| Human *CCL2* R | TCTTCGGAGTTTGGGTTTGC |
| Human *CCL5* F | CTTTGCCAGGGCTCTGTGA |
| Human *CCL5* R | GCAGTGTTCCTCCCCTCCTT |
| Human *CXCL10* F | ATTATTCCTGCAAGCCAATTTTG |
| Human *CXCL10* R | TCACCCTTCTTTTTCATTGTAGCA |
| Human *ACE2* F | GAACAGTCCACACTTGCCCA |
| Human ACE2 R | GCACTGAAGACCCATTTTGC |
| Human *TMPRSS2* F | CACGGACTGGAT TTATCGACAA |
| Human *TMPRSS2* R | CGTCAAGGACGAAGACCATGT |
| Human *Furin* F | GCTGACCAAGTTCACCCTCG |
| Human *Furin* R | GACTGGACGTGAGGGTCTTG |
| Human *neuropilin-1* F | CTCCGGACCCATACCAGAGA |
| Human *neuropilin-1* R | CCATCGAAGACTTCCACGTAGT |
| SARS-CoV-2 *Nucleocapsid* F (TaqMan) | TTACAAACATTGGCCGCAAA |
| SARS-CoV-2 *Nucleocapsid* R (TaqMan) | GCGCGACATTCCGAAGAA |
| SARS-CoV-2 *Nucleocapsid* Probe (TaqMan) | FAM-ACAATTTGCCCCCAGCGCTTCAG-BHQ1 |
| *P. aeruginosa* 16S F | GGAGAAAGTGGGGGATCTTC |
| *P. aeruginosa* 16S R | CCGGTGCTTATTCTGTTGGT |
| *S. pneumoniae* lytA F | ACGCAATCTAGCAGATGAAGCA |
| *S. pneumoniae* lytA R | TCGTGCGTTTTAATTCCAGCT |

**Supplementary Table 7. Antibodies and counterstains used in this study.**

| **Antibodies or reagents** | **Brand** | **Cat#** | **Dilution** |
| --- | --- | --- | --- |
| Anti-CC10 Antibody (E-11) | Santa cruz | sc-365992 | 1:200 |
| Anti-p63 antibody [EPR5701] | abcam | ab124762 | 1:200 |
| MUC5AC Monoclonal Antibody (45M1) | Thermofisher Scientific | MA5-12178 | 1:200 |
| Acetylated Tubulin monoclonal antibody | Sigma | T7451 | 1:200 |
| Anti-ACE2 polyclonal Antibody | abcam | ab15348 | 1:200 |
| Goat anti-Mouse IgG Secondary Antibody, Alexa Fluor 488 | Thermofisher Scientific | A-11001 | 1:1000 |
| Goat anti-Mouse IgG Secondary Antibody, Alexa Fluor Plus 647 | Thermofisher Scientific | A32728 | 1:1000 |
| Goat anti-Rabbit IgG Secondary Antibody, Alexa Fluor 488 | Thermofisher Scientific | A-11008 | 1:1000 |
| Goat anti-Rabbit IgG Secondary Antibody, Alexa Fluor 594 | Thermofisher Scientific | A-11012 | 1:1000 |
| Phalloidin-iFluor 594 Reagent | abcam | ab176757 | 1:1000 |
| DAPI Staining Solution | abcam | ab228549 | 1:1000 |
