# Supplementary material for: The establishment of COPD organoids to study host-pathogen interaction reveals enhanced viral fitness of SARS-CoV-2 in bronchi
Source: Nat Commun. 2022 Dec 10;13:7635. doi: 10.1038/s41467-022-35253-x (PMC9735280; doi:10.1038/s41467-022-35253-x)
Supplement: Supplementary file 2 — Description of Additional Supplementary Information [file 41467_2022_35253_MOESM2_ESM.pdf]

Description of Additional Supplementary Information

File name: Supplementary Video 1

Description: Functional cilia and swirling mucus in basal-out bronchial organoids

File name: Supplementary Video 2

Description: Cross-sectional µOCT video of non-diseased (left) and COPD (right) bronchial organoids

File name: Supplementary Video 3

Description: Self-rotating ‘apical-out’ bronchial organoids with outwardly oriented cilia

File name: Supplementary Data 1

Description: Summary of marker genes for single cell RNAseq cell clustering
