# Supplementary material for: The establishment of COPD organoids to study host-pathogen interaction reveals enhanced viral fitness of SARS-CoV-2 in bronchi
Source: Nat Commun. 2022 Dec 10;13:7635. doi: 10.1038/s41467-022-35253-x (PMC9735280; doi:10.1038/s41467-022-35253-x)
Supplement: Supplementary file 7 — Reporting Summary [file 41467_2022_35253_MOESM7_ESM.pdf]

nature portfolio

Corresponding author(s): Sanjay H Chotirmall, Louisa LY Chan

Last updated by author(s): Nov 9, 2022

Reporting Summary

Nature Portfolio wishes to improve the reproducibility of the work that we publish. This form provides structure for consistency and transparency in reporting. For further information on Nature Portfolio policies, see our Editorial Policies and the Editorial Policy Checklist.

Statistics

For all statistical analyses, confirm that the following items are present in the figure legend, table legend, main text, or Methods section.

n/a	Confirmed	
☐	☒	The exact sample size (n) for each experimental group/condition, given as a discrete number and unit of measurement
☐	☒	A statement on whether measurements were taken from distinct samples or whether the same sample was measured repeatedly
☐	☒	The statistical test(s) used AND whether they are one- or two-sided<br>*Only common tests should be described solely by name; describe more complex techniques in the Methods section.*
☒	☐	A description of all covariates tested
☐	☒	A description of any assumptions or corrections, such as tests of normality and adjustment for multiple comparisons
☐	☒	A full description of the statistical parameters including central tendency (e.g. means) or other basic estimates (e.g. regression coefficient) AND variation (e.g. standard deviation) or associated estimates of uncertainty (e.g. confidence intervals)
☐	☒	For null hypothesis testing, the test statistic (e.g. F, t, r) with confidence intervals, effect sizes, degrees of freedom and P value noted<br>*Give P values as exact values whenever suitable.*
☒	☐	For Bayesian analysis, information on the choice of priors and Markov chain Monte Carlo settings
☒	☐	For hierarchical and complex designs, identification of the appropriate level for tests and full reporting of outcomes
☒	☐	Estimates of effect sizes (e.g. Cohen's d, Pearson's r), indicating how they were calculated

Our web collection on statistics for biologists contains articles on many of the points above.

Software and code

Policy information about availability of computer code

Data collection	No software was used for data collection
Data analysis	All analysis performed in this study utilize a suite of published program softwares. Single cell RNA sequencing analysis was performed using Cell Ranger 6.0.2, Seurat 4.0, Ingenuity Pathway Analysis and GSEA. Cell type annotation was first analysed using Blueprint, ENCODE and the Human Primary Cell Atlas databases using celldex (1.2.0). Pseudotime analysis was performed using Monocle.<br>Bulk RNA sequencing analysis was performed using R (v4.1.0), DESeq2 (v1.34.0) and ViSEAGO. Other data was analyzed using using GraphPad Prism software (version 8.3.0). High-resolution three-dimensional Z stacks images were acquired and processed using ZEISS ZEN Microscope Software (blue edition 3.3) (Carl Zeiss).<br><br>R scripts for single-cell RNA-seq analysis : https://github.com/chenghongsheng/SC_RNAseq-airway-organoid and DOI: 10.5281/zenodo.7290276.

For manuscripts utilizing custom algorithms or software that are central to the research but not yet described in published literature, software must be made available to editors and reviewers. We strongly encourage code deposition in a community repository (e.g. GitHub). See the Nature Portfolio guidelines for submitting code & software for further information.

Data

Policy information about availability of data

All manuscripts must include a data availability statement. This statement should provide the following information, where applicable:

- Accession codes, unique identifiers, or web links for publicly available datasets
- A description of any restrictions on data availability
- For clinical datasets or third party data, please ensure that the statement adheres to our policy

Single cell RNA sequencing: Raw fastq reads from NPOs and BOs from non-diseased and COPD were aligned to the human GRCh38. p13 (hg38) reference genome. Three publicly available published bulk RNAseq datasets ((Accession numbers: GSE124180 [https://www.ncbi.nlm.nih.gov/geo/query/acc.cgi?acc=GSE124180], GSE146532 [https://www.ncbi.nlm.nih.gov/geo/query/acc.cgi?acc=GSE146532] and GSE162154 [https://www.ncbi.nlm.nih.gov/geo/query/acc.cgi?acc=GSE162154]) of cultured airway epithelial cells and/or clinical biopsies from COPD were used to compare with our scRNA dataset. The 10× single-cell RNA sequencing data generated in this study have been deposited in the GEO database under the accession code GSE186017 [https://www.ncbi.nlm.nih.gov/geo/query/acc.cgi?acc=GSE186017].
Bulk RNA sequencing: Raw FASTQ reads were mapped to the human reference genome GRCh38.p13 (hg38). The bulk RNA sequencing data generated in this study have been is deposited in the GEO database under the accession code GSE201465 [https://www.ncbi.nlm.nih.gov/geo/query/acc.cgi?acc=GSE201465].
All other relevant data supporting the key findings of this study are available within the article and its Supplementary Information files. Source data are provided in this paper.

Field-specific reporting

Please select the one below that is the best fit for your research. If you are not sure, read the appropriate sections before making your selection.

☒ Life sciences ☐ Behavioural & social sciences ☐ Ecological, evolutionary & environmental sciences

For a reference copy of the document with all sections, see nature.com/documents/nr-reporting-summary-flat.pdf

Life sciences study design

All studies must disclose on these points even when the disclosure is negative.

Sample size	No sample-size calculation was performed in this study. The sample size was depends on the availability of clinical samples donated by diseased or healthy participants. We confirmed that there are at least n=3 for both characterization and infection experiments. Single cell analysis was performed with >10,000 cells per group to access the cellular heterogeneity.
Data exclusions	No data was excluded from analysis
Replication	We aimed to include a large set of patient-derived organoids in this study. Each experiment included a minimum of 3 biological replicates per condition across multiple experiments. Number of biological and technical replicates were specified for experiments replicated and results were presented as mean +/- standard error of mean or median +/- interquartile range. All experiments were repeated at least three times (3 different experiments for organoids) showing similar results. All attempts at replication were successful, n number is described in figure legends. Single cell RNA sequencing experiments were performed once for each sample.
Randomization	Organoids derived from non-diseased donors or COPD patients were randomized to each experiments. Assortment of well-differentiated organoids of similar morphology into experimental groups was randomized for all experiments.
Blinding	Investigators were not blinded to allocation during analyses and outcome assessment owing to the automated processes used to collect and analyse results.

Reporting for specific materials, systems and methods

We require information from authors about some types of materials, experimental systems and methods used in many studies. Here, indicate whether each material, system or method listed is relevant to your study. If you are not sure if a list item applies to your research, read the appropriate section before selecting a response.

Materials & experimental systems

n/a	Involved in the study
☐	☒ Antibodies
☐	☒ Eukaryotic cell lines
☒	☐ Palaeontology and archaeology
☒	☐ Animals and other organisms
☐	☒ Human research participants
☒	☐ Clinical data
☒	☐ Dual use research of concern

Methods

n/a	Involved in the study
☒	☐ ChIP-seq
☒	☐ Flow cytometry
☒	☐ MRI-based neuroimaging

Antibodies

Antibodies used

Primary antibodies: Anti-SCGB1A1 Antibody (E-11) (1:200, Santa cruz, Cat#sc-365992), Anti-p63 antibody [EPR5701] (1:200, abcam, Cat#ab124762), Anti-MUC5AC Monoclonal Antibody (45M1) (1:200, Thermofisher Scientific ,Cat#MA5-12178), Anti-Acetylated Tubulin monoclonal antibody (1:200, Sigma, Cat#T7451), Anti-p63-α (D2K8X) XP® Rabbit mAb (1:200, Cell Signaling, Cat#13109S) and Anti-ACE2 polyclonal Ab (1:200, abcam, Cat#ab15348)

Secondary antibodies: Goat anti-Mouse IgG Secondary Antibody, Alexa Fluor 488 (1:1000, Thermofisher Scientific, Cat#A-11001), Goat anti-Mouse IgG Secondary Antibody, Alexa Fluor Plus 647 (1:1000,Thermofisher Scientific ,Cat#A32728), Goat anti-Rabbit IgG Secondary Antibody, Alexa Fluor 488 (1:1000,Thermofisher Scientific, Cat#A-11008) and Goat anti-Rabbit IgG Secondary Antibody, Alexa Fluor 594 (1:1000,Thermofisher Scientific, Cat#A-11012)

Validation

As reported by the manufacturer, each lot of antibody is quality control tested by immunofluorescence staining with flow cytometric analysis. Product details can be find on manufacturer websites:
Anti-CC10 Antibody (E-11) (Santa cruz, Cat#sc-365992): https://www.scbt.com/p/cc10-antibody-e-11
Anti-p63 antibody [EPR5701] (abcam, Cat#ab124762): https://www.abcam.com/p63-antibody-epr5701-ab124762.html
Anti-MUC5AC Monoclonal Antibody (45M1) (Thermofisher Scientific ,Cat#MA5-12178): https://www.thermofisher.com/antibody/product/MUC5AC-Antibody-clone-45M1-Monoclonal/MA5-12178
Anti-Acetylated Tubulin monoclonal antibody (Sigma, Cat#T7451): https://www.sigmaaldrich.com/SG/en/product/sigma/t7451
Anti-p63-α (D2K8X) XP® Rabbit mAb (Cell Signaling, Cat#13109S): https://www.cellsignal.com/products/primary-antibodies/p63-a-d2k8x-xp-rabbit-mab/13109
Anti-ACE2 polyclonal Ab (abcam, Cat#ab15348): https://www.abcam.com/ace2-antibody-ab15348.html

Antibodies were previously validated for immunofluorescence of human tissue either in the laboratories of ourselves or commercial suppliers. These antibodies are also validated with human lung tissues, primary cultures and cell lines by immunostaining.

Eukaryotic cell lines

Policy information about cell lines

Cell line source(s)

Vero E6 were obtained from ATCC (ATCC® CRL-1586™) and Cultrex® R-spondin1 (Rspo1) Cells was obtained from (Trevigen Cat#3710-001-K).

Authentication

Authentication was performed by the provider.

Mycoplasma contamination

Mycoplasma testing confirmed negative at regular intervals by PCR assays with species-specific primers.

Commonly misidentified lines
(See ICLAC register)

No commonly misidentified lines were used.

Human research participants

Policy information about studies involving human research participants

Population characteristics	Non-COPD (healthy) individuals<br>Subjects with normal spirometry and no prior history of COPD or any other respiratory disease (4 Males, 3 Females) were recruited. All participants were lifelong non-smokers (except for a single ex-smoker) and all participants were not on any long-term medication.<br><br>COPD<br>Patients aged > or = 45 years with stable COPD attending respiratory outpatient clinics at tertiary referral centres for routine follow-up were recruited at three hospitals across two countries as follows: Singapore General Hospital (Singapore), St Vincents Hospital (Sydney, Australia) and the John Hunter Hospital (Newcastle, Australia). COPD was defined according to the Global Initiative for Chronic Obstructive Lung Disease (GOLD) criteria. Patients with any prior history of asthma (defined by variable symptoms and expiratory airflow limitation according to the Global Initiative for Asthma guidelines; www.ginasthma.org) and those receiving long-term oral steroids, or any immunosuppressive agents were excluded.<br>All nasopharyngeal swab donors are Male. Donors of bronchial samples include 6 males and 5 females.
Recruitment	Non-COPD participants were recruited under the approved IRB at Nanyang Technological University or John Hunter Hospital. COPD participants were recruited under the approved IRBs at Singapore General Hospital (Singapore), St Vincents Hospital (Sydney, Australia) and the John Hunter Hospital (Newcastle, Australia).<br>Healthy volunteers interested were recruited in the university by our researchers. COPD patients who returned to outpatient clinics at regular terms were recruited by clinicians. No self-selection bias or other biases are present.
Ethics oversight	This study was approved by the Institutional Review Boards (IRBs) of all participating hospitals and institutions and written informed consent was obtained from all participants. Reference numbers pertaining to ethical approvals at each site was as follows: CIRB 2020/2338 (Singapore), IRB-2020-05-004 (Nanyang Technological University, Singapore), X02-0137 (The Sydney South West Area Health Service, Australia) and H-163-1205 (The Hunter New England LHD ethics committee, Australia).

Note that full information on the approval of the study protocol must also be provided in the manuscript.
